# Adsorption of Horseradish Peroxidase on Metallic Nanoparticles: Effects on Reactive Oxygen Species Detection Using 2′,7′-Dichlorofluorescin Diacetate

**DOI:** 10.1021/acs.chemrestox.0c00430

**Published:** 2021-04-15

**Authors:** Amanda Kessler, Jonas Hedberg, Sarah McCarrick, Hanna L. Karlsson, Eva Blomberg, Inger Odnevall

**Affiliations:** † KTH Royal Institute of Technology, Department of Chemistry, Division of Surface and Corrosion Science, 100 44 Stockholm, Sweden; ‡ Institute of Environmental Medicine, Karolinska Institutet, 171 77 Stockholm, Sweden; § RISE Research Institute of Sweden, Division Bioeconomy and Health, Material and Surface Design, Box 5604, SE-114 86 Stockholm, Sweden; ∥ AIMES - Center for the Advancement of Integrated Medical and Engineering Sciences at Karolinska Institutet and KTH Royal Institute of Technology, 169 27 Stockholm, Sweden; ⊥ Department of Neuroscience, Karolinska Institutet, SE-171 77 Stockholm, Sweden

## Abstract

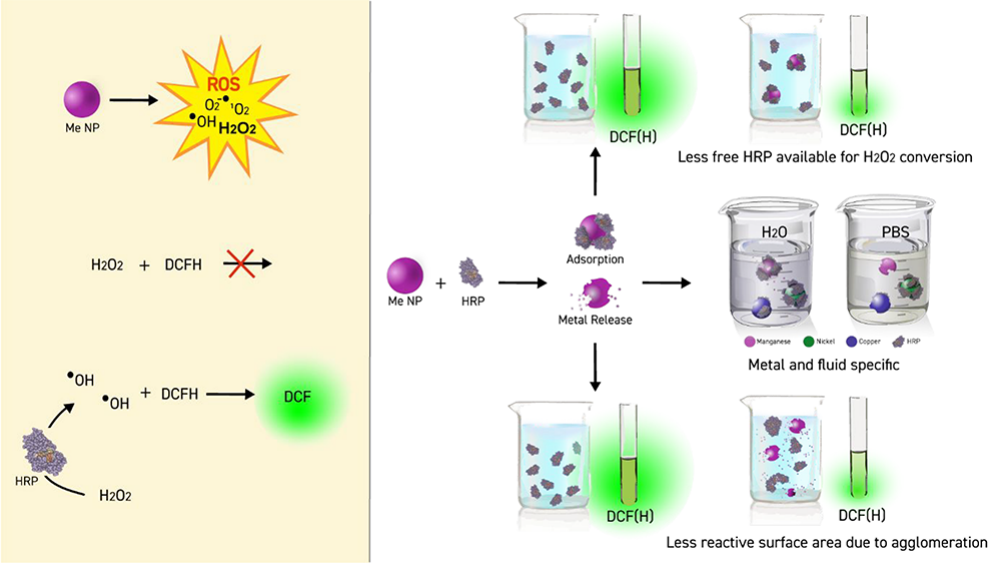

The fluorescent probe 2′,7′-dichlorofluorescein diacetate (DCFH-DA) together with the enzyme horseradish peroxidase (HRP) is widely used in nanotoxicology to study acellular reactive oxygen species (ROS) production from nanoparticles (NPs). This study examined whether HRP adsorbs onto NPs of Mn, Ni, and Cu and if this surface process influences the extent of metal release and hence the ROS production measurements using the DCFH assay in phosphate buffered saline (PBS), saline, or Dulbecco’s modified Eagle’s medium (DMEM). Adsorption of HRP was evident onto all NPs and conditions, except for Mn NPs in PBS. The presence of HRP resulted in an increased release of copper from the Cu NPs in PBS and reduced levels of nickel from the Ni NPs in saline. Both metal ions in solution and the adsorption of HRP onto the NPs can change the activity of HRP and thus influence the ROS results. The effect of HRP on the NP reactivity was shown to be solution chemistry dependent. Most notable was the evident affinity/adsorption of phosphate toward the metal NPs, followed by a reduced adsorption of HRP, the concomitant reduction in released manganese from the Mn NPs, and increased levels of released metals from the Cu NPs in PBS. Minor effects were observed for the Ni NPs. The solution pH should be monitored since the release of metals can change the solution pH and the activity of HRP is known to be pH-dependent. It is furthermore essential that solution pH adjustments are made following the addition of NaOH during diacetyl removal of DCFH-DA. Even though not observed for the given exposure conditions of this study, released metal ions could possibly induce agglomeration or partial denaturation of HRP, which in turn could result in steric hindrance for H_2_O_2_ to reach the active site of HRP. This study further emphasizes the influence of HRP on the background kinetics, its solution dependence, and effects on measured ROS signals. Different ways of correcting for the background are highlighted, as this can result in different interpretations of generated results. The results show that adsorption of HRP onto the metal NPs influenced the extent of metal release and may, depending on the investigated system, result in either under- or overestimated ROS signals if used together with the DCFH assay. HRP should hence be used with caution when measuring ROS in the presence of reactive metallic NPs.

## 1 Introduction

The use and production of engineered nanoparticles (NPs) increase with the advancements of nanotechnology. From this follows new possibilities, and applications emerge as engineered NPs have unique properties that are different from their corresponding bulk materials.^1^ Examples of applications are as components in electronics, as catalysts in paint/coating systems, and as antimicrobial additives.^2^ A reduced particle size generally means an increased total surface area for the same amount of mass, resulting in increased numbers of possible reaction sites.^3^ Such size-dependent effects are mainly observed for particle sizes <30 nm.^4^ Reactive oxygen species (ROS) production (both the intrinsic and cellular) should be evaluated since ROS generated by NPs has been shown to activate major pathways that result in DNA damage, cytotoxicity, and the development of several diseases. ROS can be generated in several different ways upon NP contact with cells. The development of new applications using engineered NPs is however far more rapid than the development of the mechanistic understanding of potential hazards and risks.^5−8^

The biouptake of metal NPs has numerous suggested reaction pathways that potentially can lead to harmful consequences for humans. NPs can affect molecular processes both passively and actively within the cell, for example, via adsorption, redox reactions, and production of ROS. As some ROS can be harmful to living organisms, this needs to be considered in nanotoxicological assessments. Since ROS production by NPs has been shown to activate major pathways that results in DNA damage, cytotoxicity and the development of several diseases,^4,9−11^ the intrinsic ROS production needs to be evaluated both intra- and acellularly. ROS can be generated in several different ways within cells upon exposure to metal NPs. One pathway is the production of radicals via Fenton-type reactions where H_2_O_2_ is transformed to HO and HO^–^.^12−14^ Another route is the production of radicals by macrophages upon initiation of NP removal.^15^ Other pathways include generation of ROS via corrosion processes^16^ and catalysis on surface oxides.^17^

**Figure 1:**
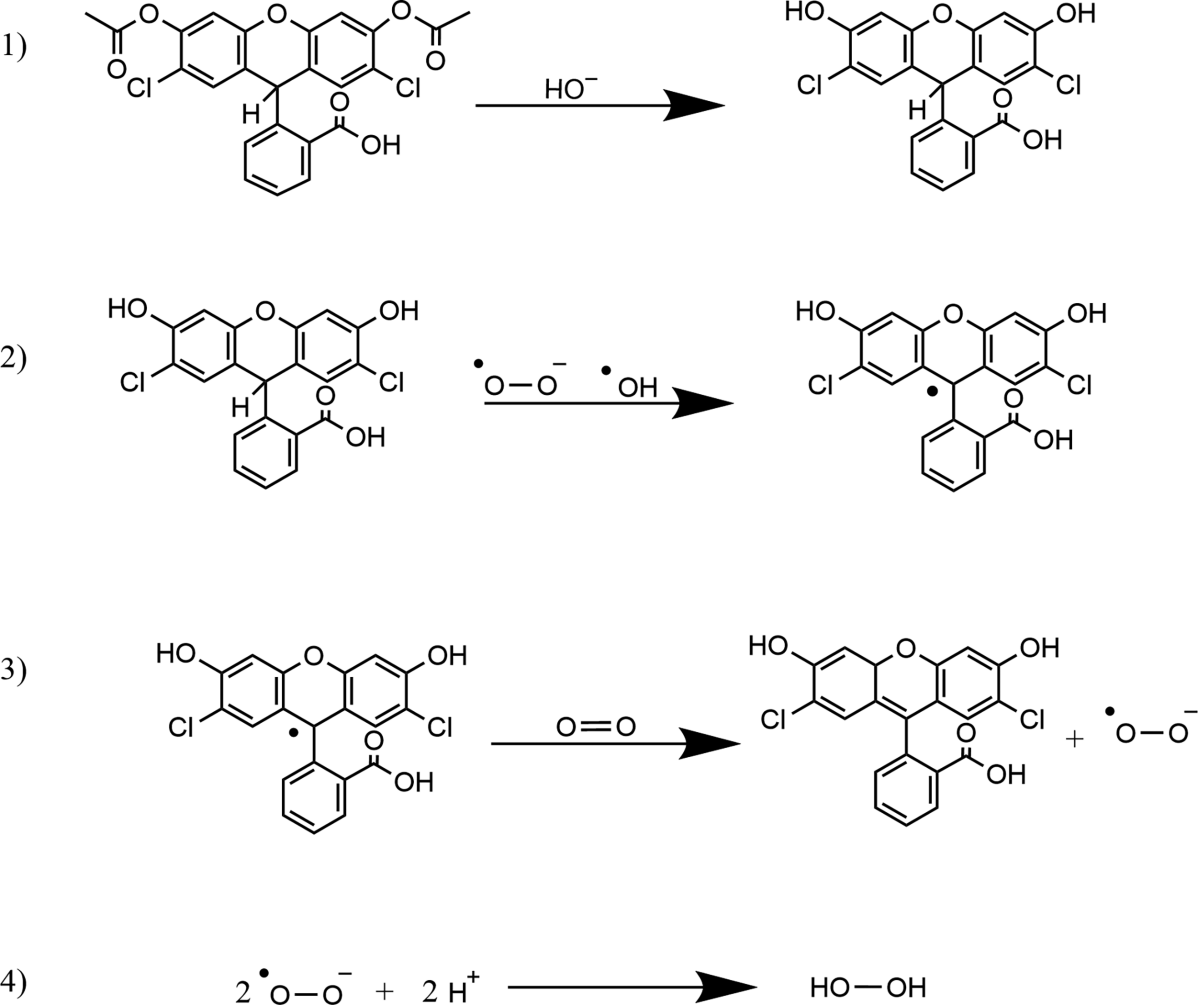
Reactions of DCFH-DA. (1) Removal of acetyl groups in alkaline solution forming DCFH. (2) ROS abstracts hydrogen forming an unstable DCF radical. (3) DCF radical reacts to fluorescent DCF with molecular oxygen forming superoxide. (4) Superoxide can react with hydrogen forming hydrogen peroxide.

The dichloro-dihydro-fluorescein diacetate (DCFH-DA) probe^18^ is a common assay to determine ROS production by NPs. For measurements in solutions without cells, acetate is often removed in alkaline solution before usage, see eq 1 in Figure 1. ROS then activates DCFH to form a reactive intermediate (eq 2), which produces the more stable fluorescent DCF compound with molecular oxygen (eq 3) forming superoxide. The superoxide can in turn react with hydrogen to form hydrogen peroxide (eq 4). In cells, deacetylation to a nonfluorescent compound takes place by cellular esterases (enzymes), which in the presence of ROS become oxidized to DCF.^18^

Since H_2_O_2_ is not reactive enough to oxidize DCFH, the horseradish peroxidase (HRP) enzyme is commonly added as it becomes activated by H_2_O_2_ and activates DCFH. Despite protocol recommendations on how to minimize false responses,^9^ this relatively simple method has been reported to sometimes generate doubtful results.^19^ Since the DCFH–HRP assay is commonly used in nanotoxicology studies today, any interactions that may lead to false responses, and hence misleading interpretations of underlying mechanisms of potentially harmful NPs need to be highlighted and avoided. The possible effect of adsorption of HRP onto the NPs and how these processes may influence the activity of HRP are less investigated in the literature. As HRP is an enzyme with an active site accessible only in one direction, its orientation and tertiary protein structure is of major importance for its activity. The adsorption of the HRP enzyme onto metallic NPs could further affect the release of metal ions, which in turn can influence its activity by, for example, partial denaturation^20^ or calcium ion (Ca^2+^) exchange.^21^ Depending on concentration, released metal ions can also influence the pH of a solution,^22^ conditions that can influence the activity of HRP which is pH dependent.^22−24^ Released metal ions in solution have moreover shown to induce biomolecule agglomeration,^25^ a process that can result in steric hindrance of the heme group of the HRP enzyme and hence reduce its activity. It is thus striking that such interactions of HRP used together with the DFCH-DA assay have not been studied in the presence of metallic NPs.

This study examined whether HRP adsorbs onto NPs of Mn, Ni, and Cu and if these processes may result in misleading results when measuring ROS production using the DFCH assay in phosphate buffered saline (PBS), saline, or Dulbecco’s modified Eagle’s medium (DMEM). The investigated NPs represent reactive particles that may induce toxic effects, partly related to ROS formation. Even though Mn is an essential element required, for example, for proper functioning of the brain as well as the nervous and enzyme systems, excess exposure has been associated with negative effects on the nervous system like cognitive and motor dysfunctions.^26−28^ Exposure to Ni NPs may increase the risk of lung damage such as lung fibrosis, respiratory tract disease, and chromosomal destruction in human lung cells and skin allergies.^29−33^ Human exposure of Cu NPs may occur, for example, through contact with inks, plastics, and particles generated in the subway.^34,35^ Cu is, similar to Mn, an essential metal for life. Exposure to Cu NPs has though been shown to increase the risk of pulmonary inflammation and to reduce the natural defense and clearance of hostile bacteria in the lungs.^36,37^

## 2 Theoretical Reaction Pathways of HRP and DCFH

HRP is an enzyme with the ability to decompose H_2_O_2_. Based on its isoelectric point (iep), between 8.7 and 9,^38^ the calculated net charge of HRP is +2 at neutral pH. The globular protein consists of 308 amino acid residues and three metal centers: the heme group and two Ca^2+^ ions. The latter are essential for the stability of its secondary structure (mainly α helix).^39,40^ A depletion of Ca^2+^ can reduce the activity of HRP with up to 40%.^21^ Since denaturation can lead to the loss of the heme group, the tertiary structure is critical for the enzyme activity.^21,39,41−43^ Since the premolten globule has a p*K*_a_ of approximately 5, melting of the tertiary structure has been observed at low pH (<4.5) and temperatures exceeding 45 °C.^39^

**Figure 2:**
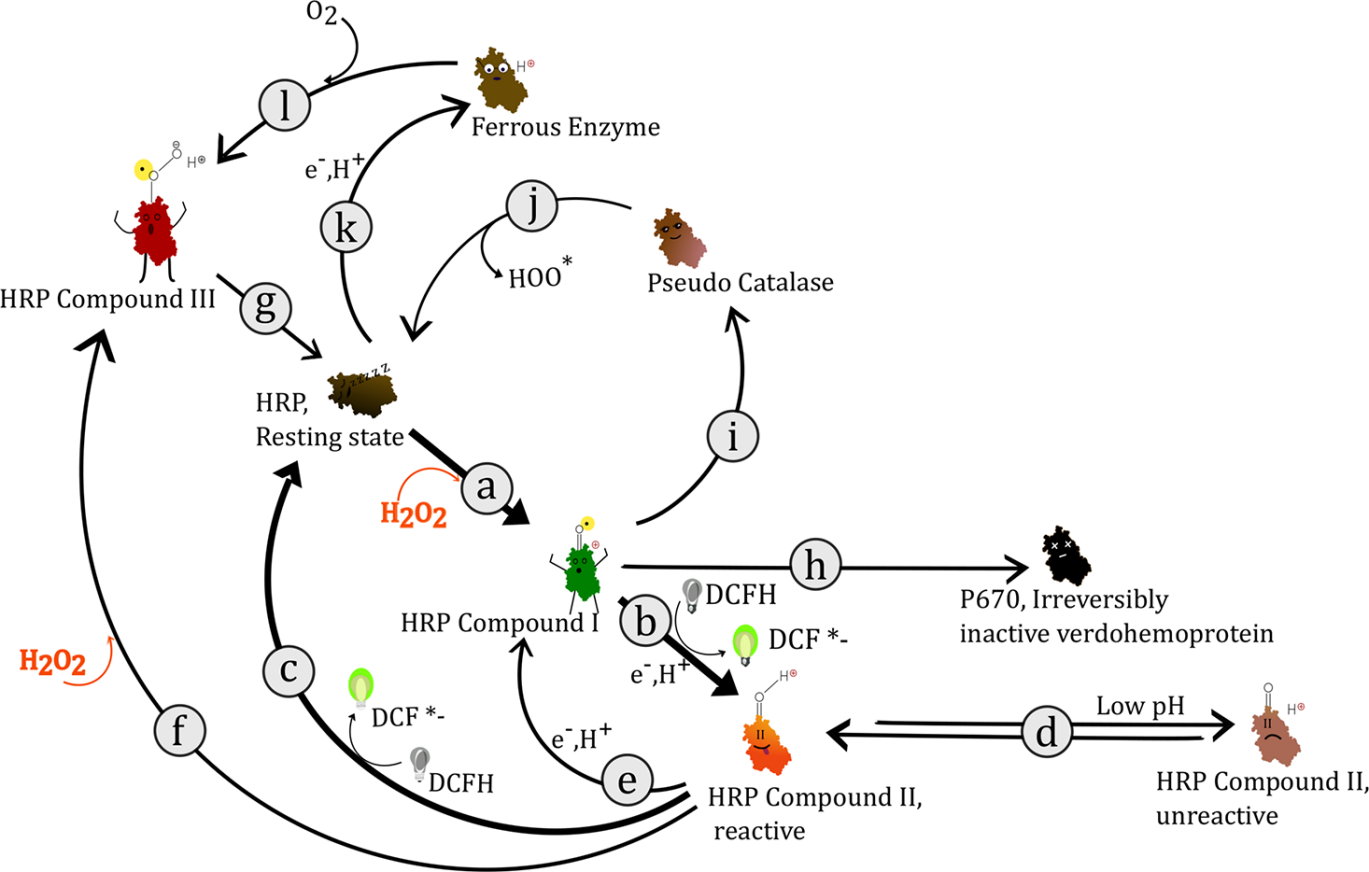
Schematic illustration of possible reaction pathways of HRP and DCFH. Each step is explained in the text.

Possible reaction pathways of HRP and DCFH are schematically summarized in Figure 2. H_2_O_2_ functions as an electron receptor in the oxidation reaction producing HRP compound I from the HRP resting state, with an oxyferryl heme group and a porphyrin π cation radical^21,41,44,45^ ((a) in Figure 2). Compound I can be reduced via electron transfer with metal and metal oxide surfaces.^45^ The reduction of compound I to compound II (b) takes place via an hydrogen-transfer reaction from the DCFH molecule, forming an intermediate DCF^•–^ radical. This radical is also produced when compound II reacts back to the resting state^46,47^ (c). Compound II is pH sensitive and active only at a lower pH (pH 4.5–7)^39^ (d). Compound II can either react back to the resting state (c), to compound I (e), or to compound III (f). Compound III has a superoxide character and is highly reactive.^42^ Compound III reacts to HRP in its resting state (g). Compound I can also irreversibly react to the inactive verdohemoprotein, P670^41^ (h), a reaction that is driven by excess H_2_O_2_.^48^ Compound I can furthermore react to pseudo catalase (i), which in turn reacts to the HRP resting state via the production of the HOO^•^ radical (j). HRP in its resting state can form a ferrous enzyme (k) which can produce compound III in the presence of molecular oxygen (l). DCFH can moreover react with reactive nitrogen species (RNS) that could be present in a cellular environment.^49,50^

## 3 Experimental Section

### 3.1 Nanoparticles

Mn and Cu metal NPs were purchased from American Elements, Los Angeles, CA, USA (average particle size <100 nm, purity >99.9%). Ni NPs (<100 nm diameter, purity >99%) were acquired from Sigma-Aldrich.

### 3.2 Solutions and Chemicals

PBS was prepared using 8.77 g/L NaCl (VWR Chemicals, AnalaR Normapur), 1.28 g/L Na_2_HPO_4_, 1.36 g/L KH_2_PO_4_ (EMSURE, anhydrous for analysis), 370 μL/L 50% NaOH (Emsure, 50%, for analysis), and ultrapure water (18.2 MΩ cm, MilliPore, Solna, Sweden. The pH was adjusted to 7.4. The ionic strength was approximatley 0.23 M.

Saline was prepared using 8.77 g/L NaCl (VWR Chemicals, AnalaR Normapur) and ultrapure water, and the pH was adjusted to 7.4 with 0.01 M NaOH. The ionic strength was approximatley 0.15 M.

DCFH-DA (Sigma-Aldrich, ≥97%) was diluted in dimethyl sulfoxide (DMSO) (Sigma-Aldrich, ReagentPlus, ≥99.5%). DMEM, without phenol red ([+] 4.5 g/L d-glucose, [−] l-glutamine, [−] pyruvate), was purchased as a sterile solution from Gibco, Thermo Fisher Scientific, Stockholm, Sweden.

Peroxidase from horseradish (Sigma-Aldrich, type II, essentially salt-free, lyophilized powder) was diluted in the specific medium of interest.

NiCl_2_, MgCl_2_ (Sigma-Aldrich, powder and chunks, ≥99% trace metal basis), and CuCl_2_ salts were used for the nanoparticle tracking analysis (NTA) investigation (see below).

### 3.3 Dispersion Preparation and Exposure in Solution

Stock suspensions of 6 mg/mL NPs in the different solutions were sonicated (USC200T ultrasonic cleaner, VWR International, bvba/sprl-B-3001, Leuven) for 10 min twice alternating with 10 s of vortexing using a vortex-genie 2 instrument (Scientific Industries, inc., Bohemia, NY, USA).

The DCFH-DA powder was dissolved in DMSO and stored in the freezer before usage. Five mM of this solution was cleaved with 0.01 M NaOH for 30 min at dark conditions, followed by dilution with a medium to which either NP stock solution or 300 u/mL HRP (1 u equals the amount of enzyme that catalyzes the reaction of one pyrogallol unit into 1.0 mg purpurogallin in 20 s at pH 6.0 at 20 °C) was added. All final samples contained 0.015 mM DCFH-DA, 1.2 mM NaOH, and 0.54 mM DMSO. The pH was adjusted to approximatley 7.4 before adding the NPs (100 mg/L). A HRP concentration of 2.2 u/mL was used for the flourescence measurements. In order to ensure its detactability, a higher concentration of HRP (8 u/mL) was used for the analyses using UV–vis, AAS, FTIR, and NTA. This HRP concentration to the NP concentration (100 mg/L) ratio (0.08) is nevertheless close to what is commonly used in nanotoxicology investigations (e.g., 2.2 u/mL HRP for a NP concentration of 25 mg/L, ratio = 0.09).

The pH was measured using a PHM210 standard pH meter (MeterLab, Radiometer analytical). All exposures were performed at 37 °C in a platform-rocker incubator at nonrocking conditions (SI80, Stuart).

### 3.4 X-Ray Photoelectron Spectroscopy

Compositional measurements of the outermost surface oxides of the different NPs (at least two different areas sized 0.3 mm^2^) were performed by means of X-ray photoelectron spectroscopy (XPS, Kratos AXIS UltraDLD, Manchester, UK) using a spectrometer equipped with a monochromatic 150W Al X-ray source. Wide and detailed spectra (pass energy 20 mV) were collected for Mn 2p, Cu 2p, Ni 2p, O 1s, and C 1s. All binding energies were adjusted in reference to the C 1s adventitious contamination peak at 285.0 eV.

### 3.5 Transmission Electron Spectroscopy

One mg/mL NPs was suspened and sonicated for 15 min (USC200T ultrasonic cleaner, VWR International, bvba/sprl-B-3001, Leuven) in 100% ethanol, followed by vortexing for 10 s. Three drops of the suspension were then applied to a 200 mesh transmission electron spectroscopy (TEM) copper grid with Formvar/carbon support films (Ted Pella, Inc.). The grids were dried and stored at ambient temperature before the measurements.

TEM images were aquired at 100 kV with magnifications of 88,000× and 18,000×.

### 3.6 Ultraviolet–vis Spectrophotometry and Atomic Absorption Spectroscopy

HRP consumption (adsorption onto NPs and/or inactivation) from solution was studied by means of UV–vis spectrophotometry (Cary 300 Bio, Varian) using a spectral bandwidth (SBW) of 4 nm, a double beam mode, a source changeover at 350.00 nm, a data interval of 1.00 nm and a scan rate of 600 nm/min. Spectra were acquired from 480 to 330 nm. Consumption of HRP due to inactivation or adsorption onto the NPs was followed by changes in peak height and position of the absorption peak at approximately 410 nm, around the Soret band. This band represents the heme group and corresponds to the tertiary structure of the enzyme.^39^ Samples were prepared without NPs and HRP as explained in section 3.3 and used for background corrections.

Prior to UV–vis analysis, exposures of the NPs were performed for 1 h at static conditions at 37 °C in a platform-rocker incubator (SI80, Stuart). The samples (three replicas for each condition) were centrifuged for 1 h at 50,000 rpm (Beckman Optima L-90K Ultracentrifuge, 1998) after which 2 mL of the centrifugate was stored (max 2 days) in dark conditions in a refrigerator. Another 2.5 mL of the centrifugate was used to quantify total concentrations of released metals. Prior to analysis by means of atomic absorption spectroscopy (AAS) equipped with a graphite furnace accessory (AAnalyst 800, PerkinElmer instruments), the samples were digested in 0.5 mL of H_2_O_2_ (30%), 50 μL of HNO_3_ (65%), and 7 mL of MQ water. Control samples were prepared using 1 mL of concentrated aqua regia and 100 μL of particle stock solution digested in a similar way (0.5 mL of H_2_O_2_ (30%), 50 μL of HNO_3_ (65%) and 8.5 mL of MQ water). Both samples and control samples were digested for 35 min using UV-light (705 UV Digester, Metrohm). Calibration standards were prepared from metal standards, 1000 μg Mn/mL (AAMN1-1), 1000 ± 5 μg Ni/mL (AANI1-1) purchased from Inorganic ventures Inc., and 2 mg Cu/L from PerkinElmer Pure. Mg(NO_3_)_2_ (PerkinElmer) and Pd(NO_3_)_2_ in 15% HNO_3_ (PerkinElmer) were used as matrix modifiers. The following standards were diluted using 1% HNO_3_ (suprapur nitric acid 65%, Merck) and ultrapure water: 10, 30, and 100 μg/L for Mn; to 5, 10, 30, 45, 100, and 300 μg/L for Ni; and to 10, 30, 100, and 300 μg/L for Cu. Mn was analyzed at 279.5 nm at temperature steps at 110, 130, 1300, 1900, and 2450 °C, Cu at 324.8 nm (110, 130, 1200, 2000, and 2450 °C) and Ni at 232.0 nm (110, 130, 1100, 2400, and 2550 °C). Argon was used for purge flow. The limits of detection for the different solutions (determined as three times the standard deviation of the background samples) were 1.5 μg/L for Mn in saline, 2.1 μg/L for Mn in PBS, 1.5 μg/L for Ni in saline, 6.1 μg/L for Ni in PBS, 3.4 μg/L for Cu in saline, and 0.8 μg/L for Cu in PBS. Quality control samples were run every sixth sample. Recovery tests using 10 μg/L for Mn, 5 μg/L for Ni, and 10 μg/L for Cu were satisfactory for all three elements: 90–110% (Mn),^51,52^ 80–110% (Ni),^53^ and 80–110% (Cu).^54^

### 3.7 Fourier Transform Infrared Spectroscopy/Attenuated Total Reflectance

Adsorption of HRP on the metal NPs was studied using Fourier transform infrared spectrometry (FTIR) using a platinum attenuated total reflectance (ATR) crystal (Bruker Tensor 37) and a flow cell. Prior to the measurements, approximately 20 drops of 3 mg/mL NP suspension (sonicated in 100% ethanol for 15 min and vortexed) were applied to the ATR crystal to create a particle film. The film was allowed to dry 2 h at ambient laboratory conditions before mounting the flow cell and initiating the analysis.

A background was retrieved with a solution of the same composition as the sample without HRP, see Section 3.3. The background solution was introduced into the flow cell ensuring that no air was trapped within the cell. After background spectrum collection, the sample solution was inserted into the flow cell. Prior to analyses and introduction of samples containing NPs, approximately 10 mL of the solution was run through the cell to ensure that the background solution completely was flushed out from the system. Both background and sample solutions were prepared simultaneously to minimize any time-related sources of error. A spectrum of the sample was acquired every 5 min up to 65 min, without any change of solution. ATR corrections^55^ and baseline corrections were applied to all spectra. In some cases, there were small signals due to water vapor in the spectra. These were removed by subtracting the appropriate amount of a spectrum of water vapor. The water vapor spectrum was collected on the ATR crystal without any sample, with the background in dry air, and the sample was collected after opening the spectrometer to the ambient air to ensure the presence of water vapor within the path of the IR beam. In order to obtain peak frequencies for the vibrational bands of interest, the ATR spectra were fitted with a Matlab routine using Gaussian peak shapes.

Compositional analyses of the NPs were performed by means of transmission FTIR with a 4 cm^–1^ resolution using a Bruker tensor 37 instrument with a MCT detector (cooled with liquid N_2_ at least 40 min before analysis). The unexposed NPs were, prior to analysis, embedded in KBr pellets (pressed at 8 bar for 2 min) and stored in a desiccator.

### 3.8 Fluorescence Measurements

Fluorescence was measured using a Infinite F200 PRO multimode plate reader from Tecan, Austria. The excitation wavelength was set to 485 nm and the emission wavelength to 535 nm. Measurements were performed every 5 min up to 65 min. Each sample was prepared in three replicates, and three separate particle suspensions were prepared for each NP. Measurements were performed using two total well volumes: 100 and 300 μL. Prior to addition of the suspensions into the wells, the pH was adjusted using 0.25% HCl. To ensure acceptable gain optimization, samples of different media with or without HRP were analyzed on separate plates. To minimize effects of natural light exposure to the samples before analysis, the lights in the fume hood in which the sample preparation was conducted were turned off and its walls covered with black plastic.

### 3.9 Nanotracking Analysis

The particle size distribution in solution was measured using NTA (NS300, Nanosight, Malvern Panalytical). Sample preparation was conducted in a dust free environment in a laminar flow cabinet. Prior to analysis, filtration (GHP Acrodisc 25 mm syringe filter with 0.2 μm GHP (polypropylene) membrane, Life Sciences) was conducted for PBS, all metal ion solutions, ultrapure water, the 5% ethanol solution, and the final HRP containg metal salt solutions. All solutions were stored at 37 °C. The HRP concentration was increased from 2.2 to 8 u/mL to increase the particle detectability. Prior to sample investigation, all equipment was rinsed with filtered 5% ethanol and dried with N_2_ gas. Three videos per sample were recorded to provide a mean value of counts and estimate the size distribution. These measurements were performed both immediately after sample preparation and after 1 h. To limit sedimentation effects, the samples were carefully mixed prior to analysis. Total metal-ion concentrations were determined using AAS as described above. Measured total concentrations of the metallic ions were 2377 μg Mn/L, 442 μg Cu/L, and 1145 μg Ni/L, and no particles were used in these experiments.

### 3.10 Metal Speciation Calculations

Visual MINTEQ 3.1^56^ and the Joint Expert Speciation System v. 8.2^57,58^ were used for chemical equilibrium calculations based on measured total concentrations of released metals, as determined by means of AAS, see above. For predictions in PBS and saline, input data of the free concentrations of the solution components were the same as in the release experiments. Some of the components of the DMEM medium (choline, pantothenate, niacinamide, inositol) were not available in the JESS database and hence not included in the calculations. Input data for the modeling are presented in Table S3 (Supporting Information) together with the free concentrations of components of the media.

## 4 Results and Discussion

### 4.1 NP Characterization

**Figure 3:**
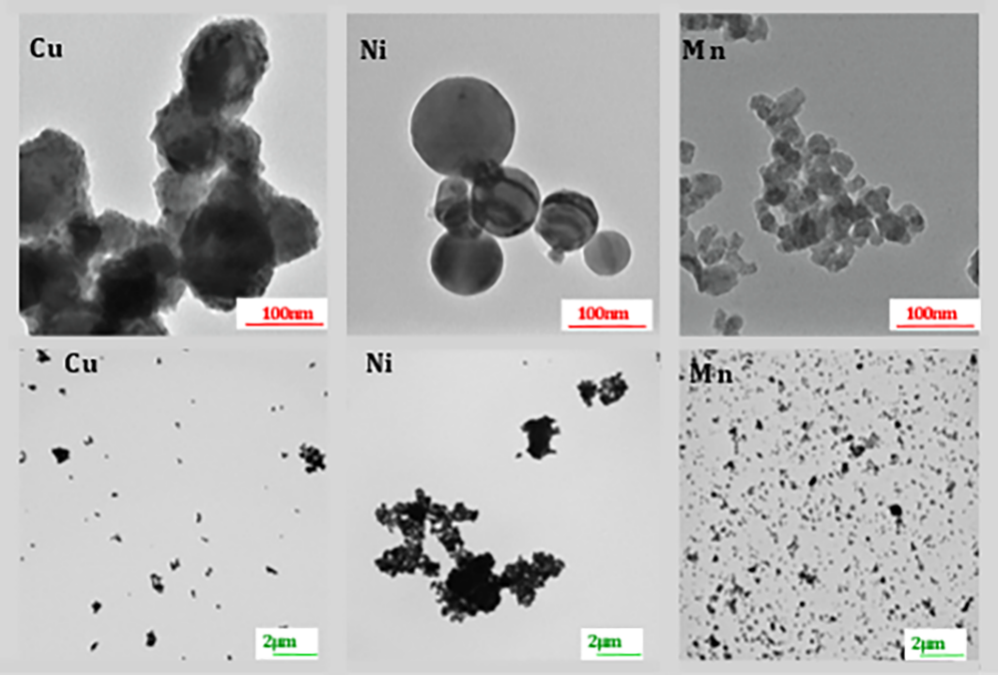
TEM images of particle shapes of the Cu, Ni, and Mn NPs at two different magnifications.

The primary particle shapes of the Cu, Mn, and Ni NPs were in all cases relatively spherical with the Mn NPs being the smallest compared with the Cu and the Ni NPs being of approximately similar size, see Figure 3 and Table 1.

**Table 1:** Primary Particle Size in Dry Conditions and Surface Composition Assessed by Means of FTIR and XPS for the Cu-, Ni- and Mn NPs

NPs	primary size (TEM) [nm]	specific surface area [m^2^/g]	surface composition (FTIR)	surface composition (XPS)
Cu	55–155	7.2^52^	Cu_2_O	CuO
Ni	55–135	6.4^31^	Ni(OH)_2_	Ni(OH)_2_, (NiO/NiOOH), Ni met^a^
Mn	15–50	26^52^	MnO/Mn_3_O_4_	MnO, Mn met^a^

a Metal signal observed indicates an oxide thickness of <5–10 nm.

According to the FTIR analysis, the Ni NPs revealed one doublet peak at 632 and 623 cm^–1^ that could be assigned to Ni(OH)_2_.^59,60^ The Mn NPs showed four peaks at 611, 507, 403, and 340 cm^–1^ indicative of the presence of both MnO and Mn_3_O_4_.^61^ Peaks at 1091, 1051, 881, 615, and 557 cm^–1^ were evident on the Cu NPs in agreement with Cu_2_O.^34,61−64^ FTIR spectra and more details are given in Supporting Information (Figure S1 and Table S1).

The presence of Ni(OH)_2_ was also indicated by the XPS findings with a major peak at 855.6 ± 0.1 eV and corresponding characteristic satellite peaks. The minor presence of NiO and/or NiOOH cannot be excluded completely, and suggested from the calculated modified Auger parameters (≈1700 and 1698 eV).^65,66^ The metallic Ni peak observed at 852.7 ± 0.1 eV indicates an oxide thickness of <5–10 nm. The XPS spectrum of the Mn NPs showed the metallic peak of Mn at 638.2 ± 0.1 eV and peaks at 640.2 ± 0.1 eV, 641.5 ± 0.2 eV, and 643.1 ± 0.1 eV that may be assigned to MnO.^52,66−68^ The XPS spectrum of the Cu NPs showed strong Cu(II) satellites and two main peaks at 933.1 ± 0.1 eV and 935.0 ± 0.1 eV that may be assigned to CuO.^52,66^ More details are given in Table S2.

### 4.2 Adsorption of HRP onto NP Surfaces

Adsorption of HRP to metallic NPs may affect the active site of HRP toward H_2_O_2_ and thus also adversely interact with the ROS measurements using the DCFH-DA assay and HRP.^69^ Adsorption of HRP has in several cases (e.g., polyaniline-composites, graphite and titanate compounds) resulted in approximately 60 to 90% lower HRP activity compared with HRP in solution.^69−71^ The reason has, for example, been attributed to denaturation of the HRP molecules or changes in their orientation, making the heme group less accessible. Large conformational changes of adsorbed HRP have also been suggested to reduce its activity.^72^

**Figure 4:**
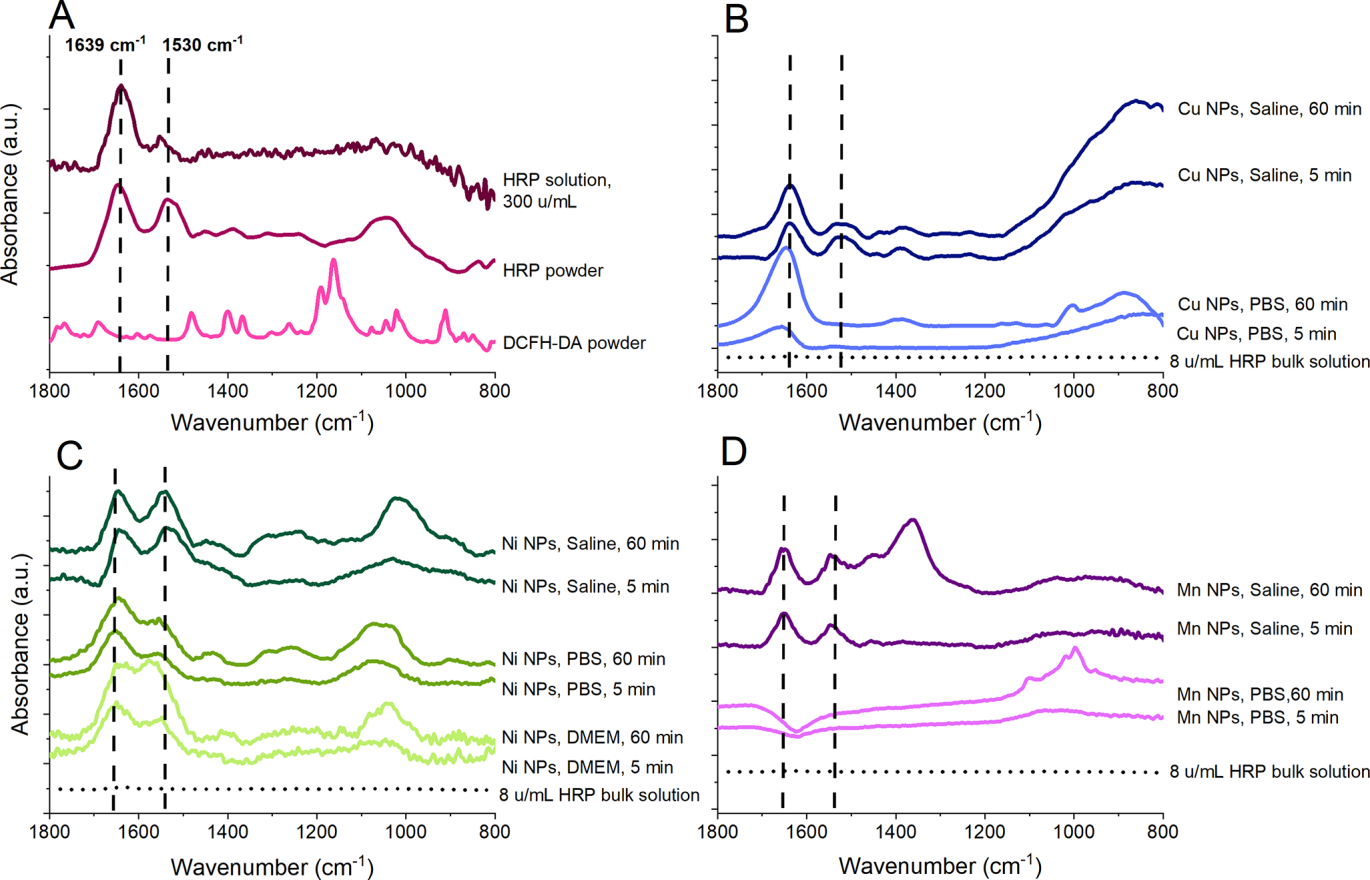
FTIR/ATR spectra after 5 and 60 min exposure in PBS, saline, and DMEM (for Ni NPs only). (A) HRP solution, HRP powder, and DCFH-DA, (B) Cu NPs, (C) Ni NPs, and (D) Mn NPs. All spectra are baseline corrected and ATR-corrected, and water vapor has been subtracted when required. The dashed lines mark the positions of the vibrational bands of amide I and amide II in HRP. The spectra have been offset for clarity, and the spectra of the HRP powder, HRP solution, and DCFH powder have all been reduced in absorbance to match the adsorption spectra of the NPs in (B–D). The dotted spectrum at the bottom in (B–D) reflects a bulk solution spectrum (no NP film) for an 8 u/mL HRP solution. Shifts in peak positions compared with the bulk solution indicate that observed peaks for the NP film measurements originate from HRP adsorbed onto the NPs and not from HRP in bulk solution.

ATR-FTIR was employed to assess if HRP adsorbs onto the Ni, Mn, and Cu NPs in saline, PBS, and DMEM (Ni NPs only). Since no peaks were observed for the solution spectra of HRP (without any NPs) at a concentration of 8 u/mL, a spectrum of a substantially higher concentration (300 u/mL) is presented in Figure 3A to visualize its characteristic peaks. Spectra for the HRP powder and the DCFH-DA powder are included for comparison. Generated spectra are presented in Figure 4B–D for the Cu, Ni, and Mn NPs exposed for different time periods in PBS, saline, and DMEM (only for Ni NPs) containing HRP (8 u/mL) and DCFH. The assignments of the main vibrational bands (fitted using Gaussian functions) are compiled in Table 2.

**Table 2:** Summary of Assignments of Main Vibrational Bands

samples	HRP amide I (cm^–1^)	HRP amide II (cm^–1^)^73^	CH_2_ bending/amino acids side chains (cm^–1^)^74^	HRP amide III/amino acids side chains (cm^–1^)^75^	HRP C–O, C–C, C–O–C (cm^–1^)^74^	asymmetric PO_4_^3–^ (cm^–1^)^76^	symmetric PO_4_^3–^ (cm^–1^)^76^	H_2_O liberation (cm^–1^)^77,78^
HRP powder	1646	1544, 1527	1480, 1450	1388	1040, 1079			
HRP solution	1638	1551						
Mn NPs, saline	1645–1650	1538–1541	1451	1365–1370				
Mn NPs, PBS						1017, 1047, 1098	997	
Ni NPs, DMEM	1650–1645	1554–1560	1412–1450	1299	1042–1045			
Ni NPs, saline	1640	1538	1433–1441	1304–1308	1008–1024			
Ni NPs, PBS	1650	1558/1510	1436	1308	1116, 1047	1116, 1047		
Cu NPs, saline	1636–1643^a^	1535, 1520,1497	1444,1432,	1386, 1292				890
Cu NPs, PBS	1655^a^	1538		1392, 1157		1005		890

a Major contribution from H_2_O bending mode at ≈1643 cm^–1^

The presence of the amide I (1645 cm^–1^) and amide II (1545 cm^–1^) bands mainly related to vibrations of C=O and C–N groups in the polypeptide linkage^74^ clearly elucidates that HRP adsorbs onto all metal NPs and exposure conditions (except for Mn NPs in PBS). The lack of bands related to DCFH (e.g., 1160 cm^–1^) implies no or very minor adsorption (less than the detection limit of the technique) of DCFH.

Adsorption of HRP onto the Cu NPs was faster (mainly within the first 5 min and only slightly increased after 1 h) and more pronounced (higher intensities of the amide bands) in saline compared with PBS (Figure 4B). This may be a consequence of phosphate adsorption in PBS observed after 1 h (emerging bands at approximately 1005 cm^–1^). The presence of water made a detailed analysis of HRP adsorption difficult due to the overlap between the amide bands and the water bending mode at 1643 cm^–1^. The broad band centered at 890 cm^–1^ was tentatively assigned to water liberations.^77^

The Ni NPs showed similar intensities of the amide bands and adsorption rates of HRP in both saline and in PBS. However, from the presence of the broad band in the region between 1050 and 1100 cm^–1^, any effects of adsorption of phosphate from PBS cannot be ruled out. No unambiguous conclusion related to the adsorption of HRP onto the Ni NPs in DMEM could be drawn since the medium contains several amino acids that also result in bands located in the region of amide I and II (1500–1650 cm^–1^). Nevertheless, the similarity between the observed spectra in DMEM, saline, and PBS indicates that HRP also adsorbs onto the NPs when immersed in DMEM.

HRP adsorption (presence of the amide bands) was evident for the Mn NPs in saline but not in PBS. Similar to the findings for the Cu NPs in PBS, the results imply adsorption of phosphate onto the Mn NPs, see Table 2. The negative band at approximately 1620 cm^–1^ could be related to the dissolution of the Mn NPs within the particle layer of the ATR crystal, a process that results in a reduced penetration depth of the infrared beam during the ATR-FTIR measurements.^79^ Similar effects have been observed for Co NPs in PBS in the presence of polypeptides.^80^

As described above, adsorption of HRP onto the NPs may influence the active site of HRP toward H_2_O_2_ and thus impede the detection of ROS using the DCFH-DA and HRP assay.^81^ Based on the similarity between the observed amide I band position compared with the position of HRP in solution (Table 2), no major conformational changes were observed upon HRP adsorption. The position of the amide I band indicates an α-helix and β-sheet secondary structure of HRP^82^ in both solution and for adsorbed HRP. Adsorption of HRP on the NPs is expected since HRP carries a weak net positively charge at pH 7.4 (iep ∼ 9) and the surface oxide of the NPs is most probably net negatively charged.^52^ Measurements of the net charge were outside the scope of this study. Forces that govern the interaction between HRP and the NPs include mainly ionic (electrostatic) interactions and van der Waals forces (strong for metal NPs).^83^ Adsorption of HRP also results in an entropic gain that originates from the release of small molecules (e.g., water and counterions) from the surfaces of both the HRP molecule and the NPs and from small structural changes of HRP during adsorption.^84−87^

### 4.3 Effect of NPS on HRP in Solution

Since the activity of the HRP molecule influences the ROS measurements, UV–vis spectroscopy was employed to investigate if the presence and dissolution of the Mn, Ni, and Cu NPs in solution would influence the activity of non-adsorbed HRP remaining in solution.

**Figure 5:**
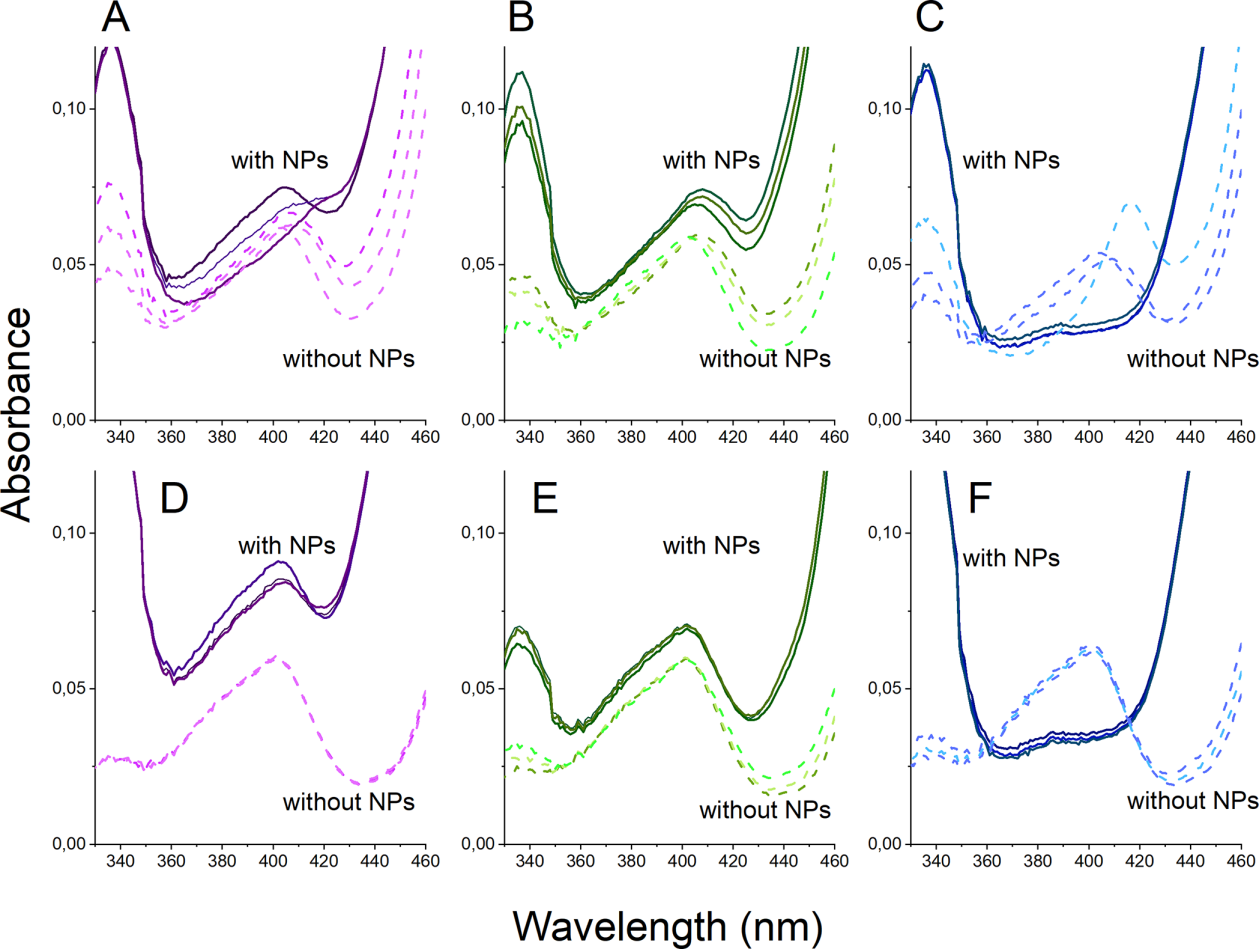
UV–vis spectra for PBS and saline solutions containing HRP (concentration 8 u/mL, pH 7.4) with and without NPs. Exposures with NPs were conducted for 1 h, followed by their removal by means of centrifugation and UV–vis analysis of the supernatant. (A) Mn NPs in PBS, (B) Ni NPs in PBS, (C) Cu NPs in PBS, (D) Mn NPs in saline, (E) Ni NPs in saline, and (F) Cu NPs in saline. Each spectrum contains three replicates with (solid lines) and without (dashed lines) NPs.

Figure 5 shows UV–vis spectra for solutions exposed with and without NPs (removed by centrifugation prior to measurements) during different time intervals.

**Figure 6:**
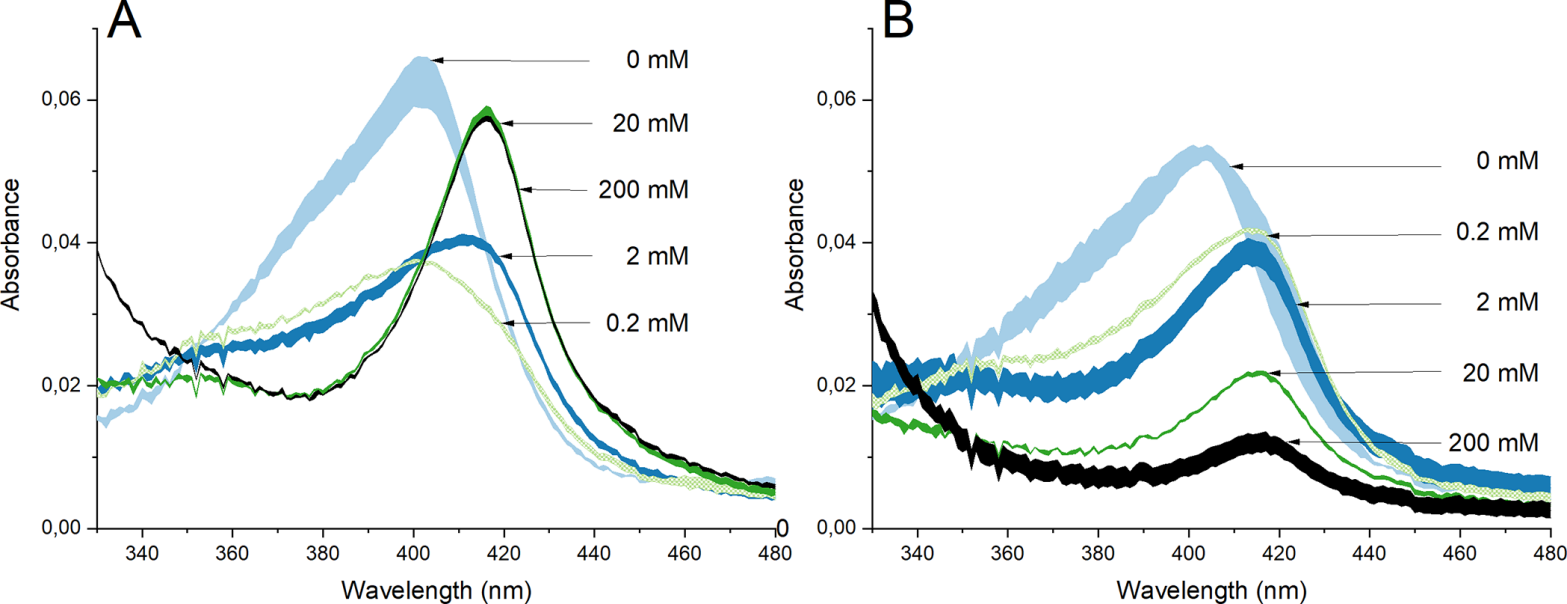
UV–vis spectra with 8 u/mL HRP in PBS (no NPs) with 0, 0.2, 2, 20, and 200 mM H_2_O_2_ measured directly after H_2_O_2_ addition (A) and after 1 h of exposure (B). Each spectrum represents mean results with standard deviations of three replicates.

The peak centered at 403 nm in the UV–vis spectra reflects the Soret band of HRP, which is closely related to its tertiary structure and activity.^47^ Reduced absorbance after 1 h was observed for all NPs and conditions, an effect most significant for the Cu NPs (Figure 5C). Generation of H_2_O_2_ from the NPs can result in changes of the state of HRP (Figure 1), in which excess H_2_O_2_ can transform HRP to compound III (with a peak at 418 nm) followed by P670, a verdohemoprotein which does not have a Soret band.^88^ One plausible explanation for the reduction in absorbance of the Soret band in the presence of NPs is the generation of H_2_O_2_, resulting in the formation of P670. This hypothesis was investigated using HRP in PBS with different concentrations of H_2_O_2_, Figure 6.

**Figure 7:**
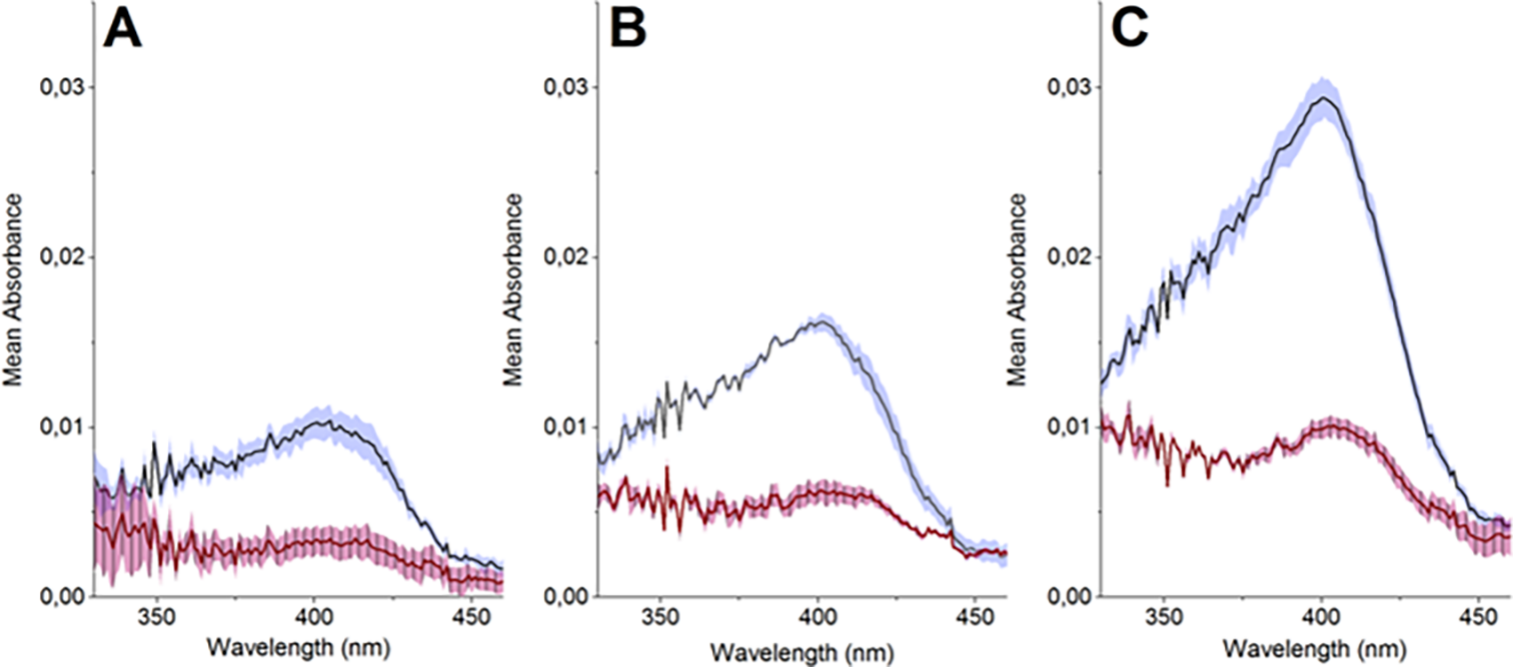
UV–vis spectra of PBS with 200 mM H_2_O_2_ and different concentrations of HRP: (A) 2 u/mL, (B) 5 u/mL, and (C) 8 u/mL measured directly after H_2_O_2_ addition. Each spectrum reflects the mean value with standard deviations of three replicates.

Figure 6A shows UV–vis spectra acquired immediately after the addition of different concentrations of H_2_O_2_ to the HRP/PBS solution. For the lowest H_2_O_2_ concentration (0.2 mM), the peak position of the Soret band remained at the same position (403 nm) but with reduced intensity. This indicates the transformation of HRP from its native state to compound I (see Figure 2).^47^ At higher H_2_O_2_ concentrations, the Soret band shifted to 417 nm, which is explained by the transformation of HRP into compound III.^88^ After 1 h of exposure to H_2_O_2_, independent of concentration, all bands had shifted to 417 nm (Figure 6B). The absorbance of the spectra of the higher H_2_O_2_ concentrations (20 and 200 mM) was in addition considerably reduced. This indicates the formation of p670, which does not have a Soret band 88 (Figure 2). As illustrated in Figure 7, the absorbance of the Soret band correlates with the concentration of HRP. As excessive amounts of H_2_O_2_ can cause formation of p670, observed in Figure 6, this means that H_2_O_2_ generation is a likely explanation for the reduction in HRP absorbance of the Soret band (Figure 5). The generation of H_2_O_2_ upon oxidation/corrosion of metals is well-known,^16,89^ for example, observed for Cu.^90^

The observed reduction in absorbance of HRP can also be related to its adsorption onto the NPs. Such strong interactions between the HRP molecules and the NPs could result in the removal of HRP during the centrifugation step. However, the relatively small extent of HRP adsorption observed by means of FTIR/ATR onto the Cu NPs in PBS compared with the other NPs (Figure 4) did not correlate to the strong loss in HRP signal observed in the UV–vis spectrum (Figure 6). The reduction in absorbance observed with UV–vis was not likely connected to any large extent to HRP adsorption on the NPs. The same argument was made for the Mn NPs in PBS that did not show any detectable adsorption of HRP in the ATR-FTIR spectra. Ion exchange between released metal ions and Ca^2+^ in HRP could also result in partial denaturation of HRP and hence in a reduced Soret band signal.^91^

In all, independent of if the reason to an observed reduction in absorbance of the Soret band in the presence of metal NPs is related to the adsorption of HRP onto the NPs or to the transformation of HRP to p670, the results indicate a loss in HRP activity. From this follows that a reduced fluorescence triggered by DCFH (Figure 2) could indicate a possible false response on actual ROS levels.

### 4.4 Effects of HRP on the Extent of Metal Release

Previous investigations have shown biomolecules in solution to both enhance and reduce the extent of metal release (ligand-induced dissolution).^14,92^ Metal release studies were therefore conducted to assess whether the presence of HRP would increase or reduce the amount of released metals from the Mn, Ni, and Cu NPs exposed in saline and PBS solutions for 1 h, both with and without HRP.

Released metal ions have been shown to influence the specific enzymatic activity of HRP in metal-specific ways which can cause activation or inhibition of HRP.^20^ One probable reason for inactivation is ion exchange with Ca^2+^.^91^ Low concentrations of Ni ^2+^ (<5 mM) and very low concentrations of Cu^2+^ (<0.3 mM) have been shown to activate HRP, while higher concentrations have shown inhibitory effects.^20^ Released concentrations of Ni and Cu ions in this study is hence expected to increase the HRP activity, while released Mn ions will act inhibitory.^20^ The strength of the inhibition and activation varies among metal ions, with Mn being the most powerful inhibitor compared with Ni and Cu.^20^

**Figure 8:**
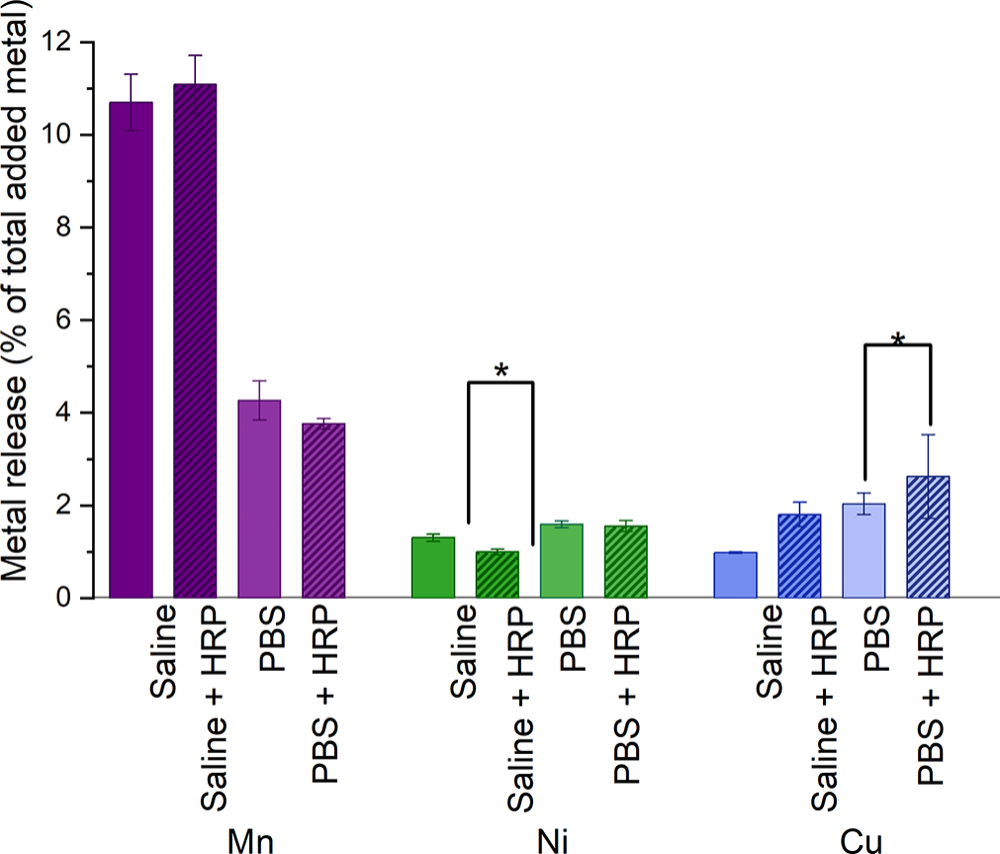
Released metal fraction (released mass/loaded particle mass) from Mn, Ni, and Cu NPs exposed for 1 h in saline and PBS, with and without HRP. Statistical analyses were performed using the Student’s *t* test. Pairs marked with * have *p*-values <0.05.

The release of metals was clearly affected by the exposure solution (PBS or saline), in particular observed for the Mn NPs (Figure 8). The presence of HRP slightly reduced the amounts of released Mn from the Mn NPs in PBS and of Ni from the Ni NPs in saline. Its presence slightly increased the amount of released Cu from the Cu NPs in both saline and PBS. Changes in metal release due to the presence of HRP were relatively small, on the order of 0.3–0.6% of the total amount of added metal (corresponding to metal concentrations of ca. 300–600 μg/L, i.e. 5–10 μM).

Theoretically, but not observed in this study, released metal ions can also affect ROS formation by, for example, Fenton-like reactions or production of ROS via electrochemical processes taking place at the NP surface. If HRP influences the extent of metal release, then it will also affect these aspects of ROS generation in a manner that is both metal NP and solution specific. These effects on ROS formation combined with effects of metal ions on the activity of HRP disable direct comparison between metal NPs of different solubility. Intrinsic differences in transformation/dissolution characteristics of metallic NPs for a given solution, even without the presence of HRP (Figure 8), indicate that the HRP activity could be altered during the ROS measurements and be both metal NP and solution specific.

Reported studies on effects of metal ions on, for example, the HRP activity and Fenton reactions described above relate to metal ions. However, as the speciation of metals is solution- (and metal-) specific, complexation can also influence the HRP activity.^20^ Equilibrium calculations of the speciation of released metals from the different NPs and solutions were performed using the Visual MINTEQ and the JESS software (see the Experimental Section). The results are presented in Figure 8 (complete list of metal species is given in Tables S4–S6).

**Table 3:** Input Data for Metal Speciation Modeling Using Visual MINTEQ and JESS on metal concentrations (μg/L) of released Mn, Ni, and Cu in saline, PBS, and DMEM with and without HRP^a^

released metal concentrations (μg/L)	saline	PBS	DMEM
Mn	10313	1980	1500
Mn + HRP	7763	2377	
Ni	1145	1177	1500
Ni + HRP	1003	928	
Cu	938	420	1500
Cu + HRP	1976	442	

a Data in saline and PBS are derived from the release measurements, whereas the metal ion concentrations used for the prediction in DMEM are approximated to be in the same range as in saline and PBS.

Small changes in released metal concentrations due to the addition of HRP (Figure 8) did not influence the speciation of the released metals. Effects of the HRP concentration in solution on the metal speciation could not be predicted due to a lack of reference data in JESS database. It is speculated that HRP, similar to other biomolecules such as, for example, BSA,^93^ increases the extent of metal complexation in solution.

Released Mn was predicted to predominantly exist as free ions and labile (easily dissociated complexes) complexes in saline (95%), whereas solid MnHPO_4_ was the predominant predicted species in DMEM and PBS. Free ions (98%) and labile complexes were also predicted for released Ni in saline. Predictions of released Ni from the Ni NPs in PBS revealed strongly bonded phosphate complexes (51%) in addition to several labile complexes. Strongly bonded complexes with amino acids were the main species predicted in DMEM. Released Cu into saline was predominantly foreseen to exist as a solid phase (CuO) without any free ions or labile complexes. A similar speciation was predicted in PBS together with strong phosphate complexes. Strongly bonded complexes with amino acids were the predominant Cu species in DMEM.

**Figure 9:**
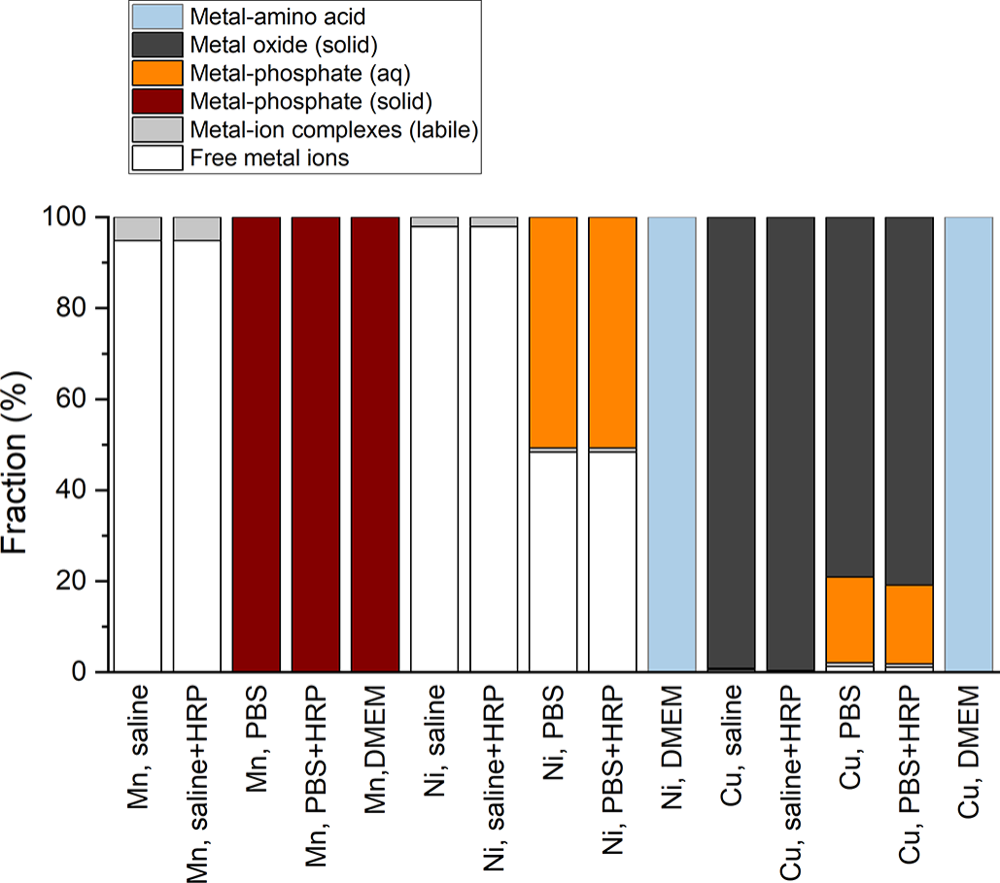
Metal speciation predictions using Visual MINTEQ and JESS for released Mn, Ni, and Cu from the different NPs in saline and PBS (with and without HRP, Table 3 and Figure 7) and in DMEM (without HRP). HRP was not included in the speciation calculations due to lack of data in the JESS software database.

The speciation of released metals in solution is important as free ions and labile species have a higher probability to interact with HRP compared with strong complexes and solid phases. This stronger interaction with HRP can possibly reduce its activity and thereby affect the ROS measurements when using the DCFH assay. Possible ROS formation via Fenton reactions may further be suppressed in the presence of free metal ions. Differences in metal speciation between different solutions (Figure 9) can thus lead to different interactions with HRP, which in turn can change the HRP activity. The speciation prediction implies that these effects could be of larger importance for, for example, Mn and Ni in saline but not in either PBS or DMEM.

The speciation predictions of solid species formation are further in agreement with the adsorption findings for the Mn NPs in PBS (Figure 5). Adsorbed phosphate species (no HRP) onto the Mn NPs could originate from the predominance and precipitation of solid MnHPO_4_. This adsorption resulted in a reduced extent of released Mn (Figure 9). Some HRP was rapidly adsorbed (within 5 min) onto the Cu NPs in PBS (Figure 5). This adsorption process was hindered with time (up to 1 h), possibly as a result of CuO formation and precipitation onto the Cu NP surfaces. This formation did not, however, result in any reduced amounts of released Cu (Figure 9).

### 4.5 Effects of Background Solution Kinetics, Solution Volume on the HRP Activity, and Fluorescence of DCFH with and without NPS

**Figure 10:**
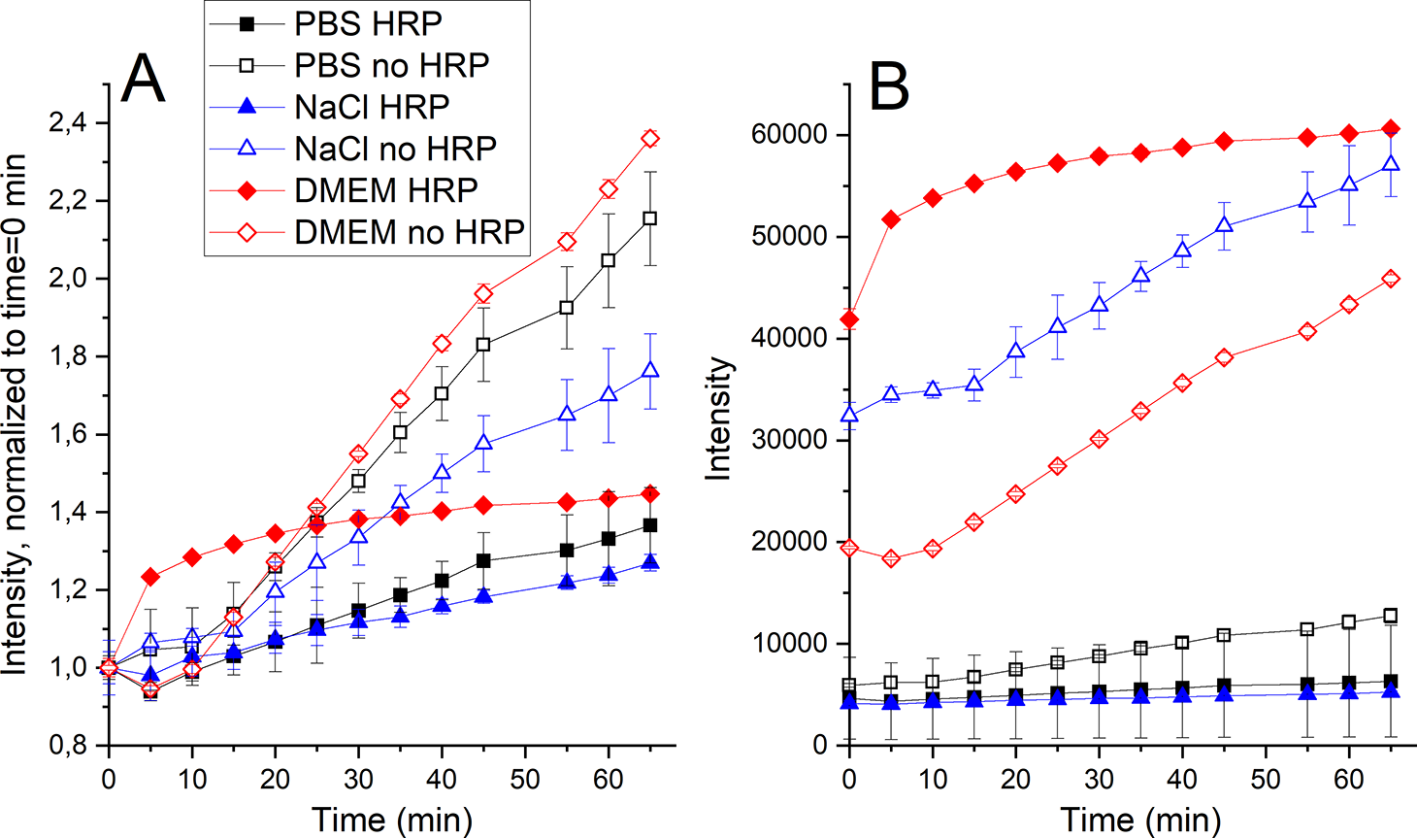
Fluorescence from DCFH in PBS, saline, and DMEM, with or without the presence of HRP (8 u/mL). (A) Intensity values normalized to the absorbance of the first time point for each solution and (B) non-normalized intensity. Since a higher gain allows for higher sensitivity, a high gain is generally used for weakly fluorescent samples, while the gain is lowered for highly fluorescent samples to avoid overload. The error bars represent the standard deviation based on three independent samples.

H_2_O_2_ cannot, as discussed above, oxidize DCFH without the presence of HRP. However, the reverse process can take place, that is, HRP can oxidize DCFH without the presence of H_2_O_2_ (Figure 2). These effects were investigated in saline, PBS, and DMEM without any NP addition (Figure 10).

**Figure 11:**
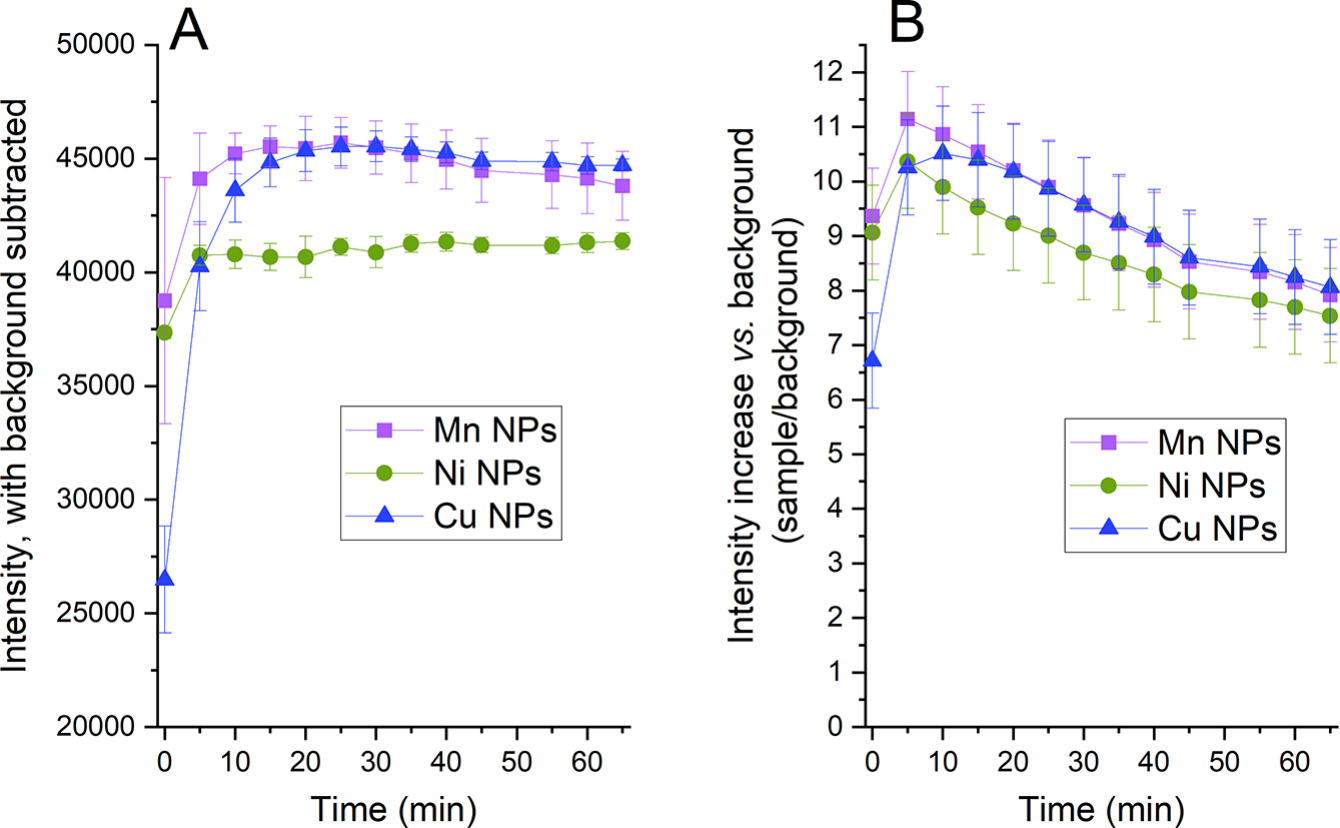
Fluorescence detected using the DCFH-DA assay with HRP (8 u/mL) in PBS in the presence of metal NPs. (A) Observed intensities subtracted with the background signal (no NPs). (B) Observed intensities divided with the background signal. The error bars represent the standard deviation of three independent samples.

The fluorescence intensity increased with time in all solutions both with and without HRP (Figure 10A). An increased absorbance with time is for the non-HRP-containing media related to self-initiation of DCFH. Due to rapid kinetics between DCFH and HRP reaching an equilibrium early in the experiment, the increase with time was slower in the presence of HRP. When comparing solutions with and without HRP, the results showed different kinetics when normalized to the fluorescence measured at time zero (0 h) (Figure 10B). The background kinetics therefore needs to be considered when comparing ROS results (both with and without HRP) in order to ensure that any observed ROS formation really reflects conditions induced by, for example, NPs. This is illustrated in Figure 11 for the Cu, Mn, and Ni NPs exposed in PBS with HRP (8 u/mL) up to 65 min. The results are presented with the background subtracted (Figure 11A) and divided (Figure 11B) with the observed intensities for solutions with NPs. The results show that both the kinetics of the background and the way of normalizing fluorescence data influence the interpretation of the measurements. For example, the fluorescence seems to decrease with time when dividing with the background, whereas it remains relatively constant after 10–20 min if subtracting the background.

It should be noted that since effects of adsorption of HRP, metal release, and metal speciation also influence the results presented in Figure 11, any detailed interpretation and/or direct comparison between the metal NPs is disabled.

**Figure 12:**
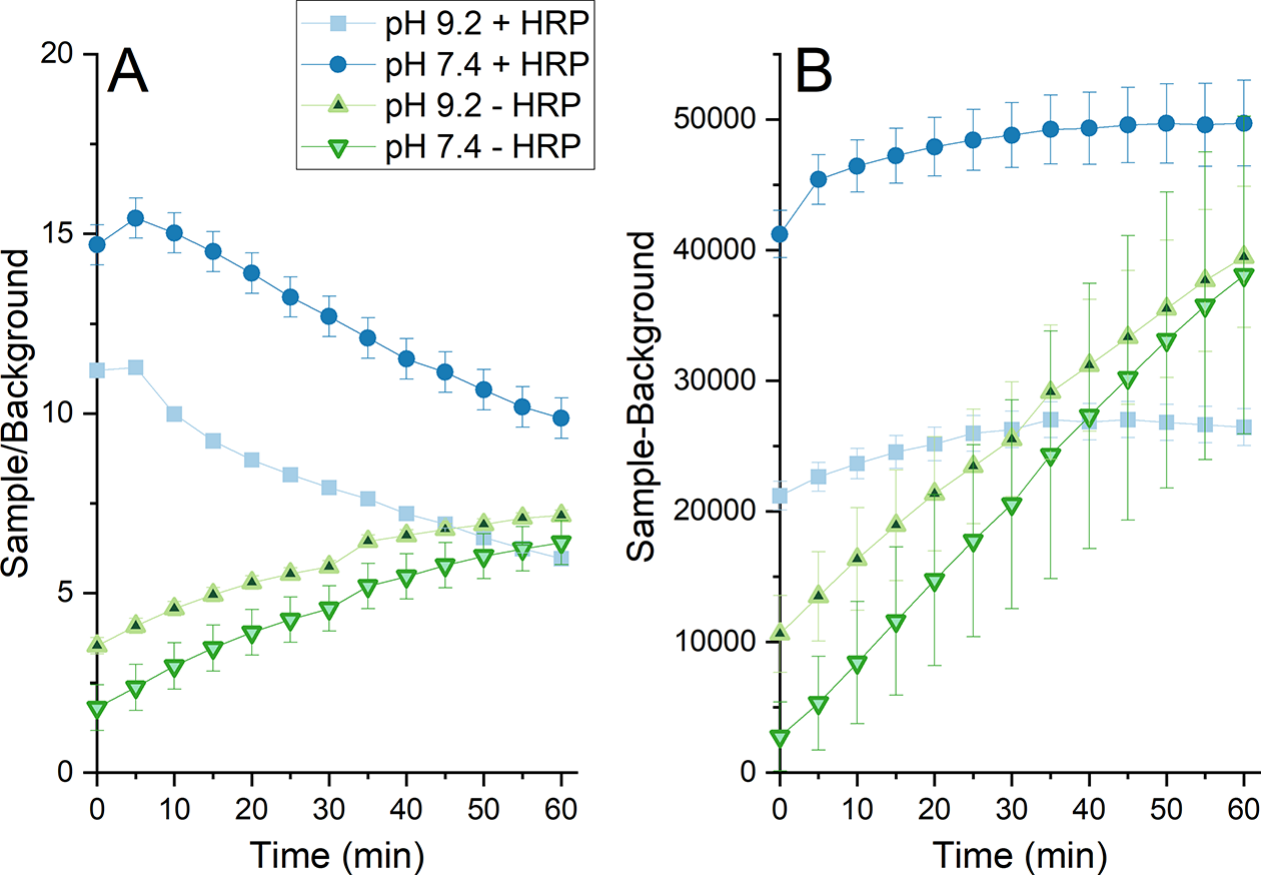
Change in ROS signal (fluorescence) determined with DCFH with (+) and without (−) HRP for nonadjusted (pH 9.2) and pH adjusted conditions (pH 7.4) with Cu NPs (100 mg/L) in PBS. The results are presented as mean values (triplicate measurements, three readings for each sample) either divided (A) or subtracted (B) with the corresponding mean value of the background (no NPs) measured for each time point in parallel.

Figure 12 shows fluorescence intensities normalized to the background for Cu, Ni, and Mn NPs in saline, PBS, and DMEM. Since the background solutions increased in intensity over time to a larger extent than conditions with NPs, observed intensities (ROS signal) seem to decrease over time. From this follows an evident risk to underestimate the ROS levels for the investigated system. This could be avoided if the fluorescence is measured after several time points in order to assess the kinetics and relevant time point to determine the ROS level for the investigated system. The use of different sample volumes for the ROS measurements resulted in different sample–air interphase areas, schematically visualized in Figure S2. A smaller volume results in a larger solution/gas surface area compared to a larger volume, and the latter can also experience a larger interference of external light. This can result in different readings during the fluorescence measurements and hence in different ranking in ROS formation (normalized fluorescence) between the NPs in PBS (see Figure S3). The underlying reason is unclear, though it highlights the importance to also report the well volume when measuring ROS using fluorescence.

### 4.6 Effects of pH, Solution, and Time on the HRP Activity

The activity of HRP has a well-studied pH dependence.^23,24^ Changes in solution pH due to, for instance, the release of metals, precipitation processes, HRP interactions with NPs, or other effects can thus result in changes in the HRP activity. Since one effect of pH is already induced upon the addition of DCFH, as it is present in an alkaline NaOH solution, all results were generated at a pH adjusted to 7.4. The measurements clearly showed a substantial change in pH upon the addition of DCFH increasing from 7.4 to approximately 10.5 in saline and from 7.4 to 9.2 in PBS. From these changes in pH follow inevitably a change in the net charge of the HRP molecule (zero at pH 9), conditions that most likely change its interaction (adsorption) with the NPs.^39^ Such effects were not investigated in this study. It is anticipated that many reported studies using the DCFH-DA + HRP assay have not taken this pH effect into account. This is most probably a consequence of scarce information available in operational protocols on how to correct for changes in solution pH after the addition of the NPs and DCFH.^94^ Differences in fluorescence from DCFH as a result of a non-adjusted solution pH are illustrated in Figure 12 for the Cu NPs exposed in PBS at pH 9.2 (nonadjusted) and 7.4 (adjusted with HCl after addition of DCFH and NaOH).

Effects of pH on the fluorescence reading were considerably larger in the presence of HRP showing a higher signal at pH 7.4 (4.2 times after 15 min) than pH 9.2 (3.4 times after 15 min). The trend over time was the same for both pH conditions. Similar signal-to-blank ratios were obtained for conditions without HRP.

The solution pH was measured before and after 1 h of exposure of the NPs in both PBS and saline (initially adjusted to pH 7.4). However, these effects on pH were relatively small with pH shifting 0.3–0.4 pH units in PBS and 0.1–0.4 units in saline.

In all, the results elucidate the importance to measure the solution pH of the DCFH + HRP solution both prior to and after exposure and emphasize the necessity to adjust the pH, when needed, to the intended pH of the medium. The results further clearly show the importance of considering the time dependence, which is both metal and solution specific.

As illustrated by the speciation modeling results (see Figure 9), the speciation of released metal ions (complexes and compounds in the presence of different ligands such as inorganic ions and biomolecules (e.g., proteins, amino acids, enzymes, etc.)) depends on the chemistry of the solution. Such metal–ligand processes influence not only the surface reactivity and chemistry of the NPs but can also result in conformational changes of both the NPs and formed species in solution, which can result in agglomeration^25^ and reduced stability (precipitation/sedimentation). An increased ionic strength of the solution due to released metal ions can in addition lead to shielding of electrostatic forces, which can cause particle agglomeration and sedimentation. Similar effects can also be observed for metal NPs as a result of strong van der Waal forces.^95^

NTA measurements were performed in PBS with soluble metal salts in order to assess whether released metal concentrations would induce any HRP agglomeration followed by precipitation, that is, conditions which would reduce the HRP activity. No evident size differences (50% of the observed particles (D50) typically varying between 100 and 170 nm or lower) were observed in PBS either with or without HRP, see Figure S4. Since these sizes are considerably larger than the size of the free HRP molecule (approximate size 4 × 6.7 × 11.7 nm),^96^ the results suggest that the measured particle size distribution in both cases (with and without HRP) reflects the background concentration rather than any agglomeration. It was further concluded that the given metal ions and their concentrations did not induce any agglomeration of HRP, though it is possible that any effect of metal ions on HRP could be hidden due to low particle counts and large particle size spans.

## 5 Concluding Remarks

Possible false responses on measured ROS levels induced by the adsorption of horseradish peroxidase (HRP) onto Mn, Ni, and Cu NPs and concomitant effects of released metals when exposed in synthetic biological fluids including phosphate buffered saline (PBS), saline, and Dulbecco’s modified Eagle’s medium (DMEM) were investigated using the fluorescent probe 2′,7′-dichlorofluorescein diacetate (DCFH-DA). The following remarks could be made: HRP adsorbed onto most of the NPs, with less adsorption taking place for the Cu and Mn NPs in PBS. HRP influenced the metal release process for the Ni NPs in saline and for the Cu NPs in PBS. Since both released metal ions and adsorption of HRP onto the NPs can result in a changed activity of HRP, this may lead to a false response of the ROS level measured by means of DCFH combined with HRP. The assay gives a signal if there is ROS present in the system or not, but its magnitude can either be under or overestimated, and the results of different exposure conditions are difficult to compare. Observed differences in metal speciation of released metals from the NPs influence the HRP activity, and thus the measured ROS levels.

The solution pH changes due to the addition of alkaline medium during preparation of DCFH. From the high pH sensitivity of HRP follows an evident risk of loss in its activity, which in turn influences measured ROS levels. Since both the presence of metallic NPs and the release of metals can change the solution pH, the solution pH must be monitored closely. Furthermore, the results display the importance to consider the kinetics of the background (faster in the presence of HRP) when normalizing fluorescence data.

Due to adsorption of HRP onto the reactive metal NPs of this study (Mn, Ni, and Cu NPs) and its influence on the metal release process, ROS measurements in synthetic fluids may result in false ROS responses. These aspects as well as possible surface and solution interactions should be considered when using the DCFH and HRP assay for ROS studies with metal NPs.

## Supporting Information Available

Figure S1: FTIR transmission spectroscopy on the Ni, Cu and Mn NPs embedded in KBr pellets. Table S1: Summary and assignment of main vibrational bands in Figure S1. Table S2: Summary of XPS results. Table S3: Metal ion concentrations (Mn^2+^, Ni^2+^, Cu^2+^) used in the metal speciation calculations. Table S4: Equilibrium calculations results for Mn in solution. Table S5: Equilibrium calculations results for Cu in solution. Table S6: Equilibrium calculations results for Ni in solution. Figure S2: Schematic illustration of sample solution in a well for fluorescence measurements. Figure S3: Fluorescence measurements of Cu, Ni, and Mn NPs dispersions in NaCl, PBS, and DMEM with and without HRP (2.2 u/mL) using two well volumes. Figure S4: Particle size distributions determined by NTA for metal added to PBS + HRP (PDF)
