# Supplementary material for: Adsorption of Horseradish Peroxidase on Metallic Nanoparticles: Effects on Reactive Oxygen Species Detection Using 2′,7′-Dichlorofluorescin Diacetate
Source: Chem Res Toxicol. 2021 Apr 15;34(6):1481–95. doi: 10.1021/acs.chemrestox.0c00430 (PMC8220500; doi:10.1021/acs.chemrestox.0c00430)
Supplement: Supplementary file 1 — tx0c00430_si_001.pdf [file tx0c00430_si_001.pdf]

Characterization of interactions between horseradish peroxidase and metallic nanoparticles – implications for detecting reactive oxygen species using 2',7'-dichlorofluorescin diacetate

Amanda Kessler,[a,*] Jonas Hedberg,[a] Sarah McCarrick,[b] Hanna Karlsson,[b] Eva Blomberg,[a, c] Inger Odnevall [a,*]

[a] KTH Royal Institute of Technology, Department of Chemistry, Div. Surface and Corrosion Science, Stockholm, Sweden

[b] Institute of Environmental Medicine, Karolinska Institutet, Stockholm, Sweden

[c] RISE Research Institutes of Sweden, Division Bioeconomy and Health, Material and Surface Design, Stockholm, Sweden

[*] Corresponding authors, email: akessler@kth.se or ingero@kth.se

Table S1. Summary of XPS results.

**Binding energy (eV)**	**Peak**	**Assignment**
Mn NPs		
641.3	Mn 2p3/2	$Mn_3O_4/MnO_2/Mn_2O_3/MnOOH$ [1]
638.6	Mn 2p3/2	Mn(0) [2]
530.0	O 1s	Mn(II)/Mn(III)/Mn(IV) [2]
532.1	O 1s	Mn(II)/Mn(III)/Mn(IV) [2]
533.7	O 1s	Mn(II)/Mn(III)/Mn(IV) [2]
Ni NPs		
852.7	Ni 2p3/2	Ni(0) [2]
854.1	Ni 2p3/2	Ni_2O_3/NiOOH/ $Ni(OH)_2$ [2]
855.7	Ni 2p3/2	NiOOH/$Ni(OH)_2$/NiO [2]
858.0	Ni 2p3/2	NiOOH/ $Ni(OH)_2$ [2]
860.3	Ni 2p3/2	$Ni(OH)_2$ [2]
862.6	Ni 2p3/2	$Ni(OH)_2$ [2]
529.5	O 1s	Ni(II) [2]
531.6	O 1s	Ni(II) [2]
533.4	O 1s	Ni(II) [2]
Cu NPs		
933.1	Cu 2p3/2	Cu(0)/Cu_2O [3]
934.8	Cu 2p3/2	CuO/$Cu(OH)_2$ [1, 3]
940.7	Cu 2p3/2	CuO/$Cu(OH)_2$ [1, 3]
943.5	Cu 2p3/2	CuO/$Cu(OH)_2$ [1, 3]
529.8	O 1s	CuO [3]
531.7	O 1s	CuO/$Cu(OH)_2$ [3]

Infrared spectroscopy

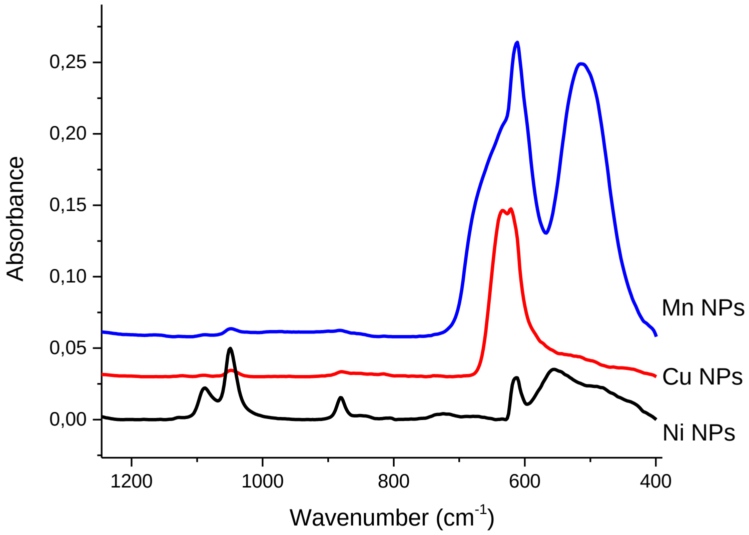

Figure S1. FTIR transmission spectroscopy on NP powder. The NPs were embedded in a KBr pellet**.**

Table S2. Summary and assignment of main vibrational bands in Figure S1.

**Particle**	**Peak position (cm^{-1})**	**Assignment**
**Ni**	487	$Ni(OH)_2$ [4, 5] ([6]
**Ni**	554	$Ni(OH)_2$ [4, 5, 7] ([6]
**Ni**	612	$Ni(OH)_2$ [6, 8]
**Ni**	879	
**Ni**	1049	CO_2/CO_3 [7]
**Ni**	1090	
**Mn**	611	Mn_3O_4[9, 10] [11, 12]
**Mn**	507	Mn_3O_4[9, 10] [11, 12]
**Mn**	403	Mn_3O_4[9, 10]
**Mn**	340	Mn_3O_4[9, 10]
**Cu**	635	Cu_2O [13]

Metal speciation calculations

Table S3. Metal ion concentrations used in the metal speciation calculations.

Solution	Concentration
Mn NaCl HRP	10 313 µg/L Mn^{2}
Mn NaCl	7763 µg/L Mn^{2+}
Mn PBS	1980 µg/L Mn^{2+}
Mn PBS+HRP	2377 Mn^{2+}
Mn DMEM	1500 µg/L Mn^{2+}
Ni NaCl + HRP	928 µg/L Ni^{2+}
Ni NaCl	1003 µg/L Ni^{2+}
Ni PBS	1177 µg/L Ni^{2+}
Ni PBS+HRP	1145 µg/L Ni^{2+}
Ni DMEM=	1500 µg/L Ni^{2+}
Cu NaCl+HRP=	1976 µg/L Cu^{2+}
Cu NaCl	938 µg/L Cu^{2+}
Cu PBS	420 µg/L Cu^{2+}
Cu PBS + HRP	442 µg/L Cu^{2+}
Cu DMEM	1500 µg/L Cu^{2+}

Table S4. Equilibrium calculations results for Mn ions in solution.

**Mn, saline**	**Mn, saline, HRP**	**Mn, PBS**	**Mn, PBS, HRP**
94.8% Mn^{2+}	94.8% Mn^{2+}	99.999% $MnHPO_4$(s)	99.999% $MnHPO_4$(s)
4.5% $MnCl^{+}$	4.5% $MnCl^{+}$	0.00074% $MnHPO_4$(aq)	0.00074% $MnHPO_4$(aq)
0.64% $MnCl_2^{-}$	0.64% $MnCl_2^{-}$	0.00024 Mn^{2+}	0.00024 Mn^{2+}

Table S5. Equilibrium calculations results for Cu ions in solution**.**

**Cu, saline**	**Cu, saline, HRP**	**Cu, PBS**	**Cu, PBS, HRP**
99.1% CuO(s)	99.6% CuO(s)	79.1% CuO(s)	80.2% CuO(s)
0.52% Cu^{2+}	0.23% Cu^{2+}	18.9% $CuHPO_4$(aq)	17.2% CuHPO§(aq)
0.31% $CuOH^{+}$	0.14% $CuOH^{+}$	1.2% Cu^{2+}	1.1% Cu^{2+}
		0.83% $CuOH^{+}$	0.67% $CuOH^{+}$

Table S6. Equilibrium calculations results for Ni ions in solution. Tyr=Tyrosine, Cys=Cysteine, His=Histidine

**Ni, saline**	**Ni, saline, HRP**	**Ni, PBS**	**Ni, PBS, HRP**	**Ni, DMEM**
97.9% Ni^{2+}	97.9% Ni^{2+}	50.5% $NiHPO_4$(aq)	50.5% $NiHPO_4$(aq)	65%. $NiH_6Tyr_3^{2+}$
1.8% $NiCl^{+}$	1.8% $NiCl^{+}$	48.1% Ni^{2+}	48.1% Ni^{2+}	12%. $NiH_4Tyr_2^{2+}$
0.3% $NiOH^{+}$	0.3% $NiOH^{+}$	0.85% $NiCl^{+}$	0.85% $NiCl^{+}$	7%. $NiCys_2^{2-}$
				5%. $NiHisCys^{-}$
				5%. $NiHis_2$
				3% NiH_5Tyr^{+}
				1% $NiGlnCys^{-}$
				1% NiGlnHis

Table S7. Detection limit of AAS quantification for Mn, Ni, Cu. The detection limit was calculated from mean of 3 times the standard deviation of blank samples.

Sample series	Detection limit ug/L
**Mn NaCl**	0.5
**Mn PBS**	2.1
**Ni NaCl**	1.5
**Ni PBS**	6.1
**Cu NaCl**	3.2
**Cu PBS**	0.8

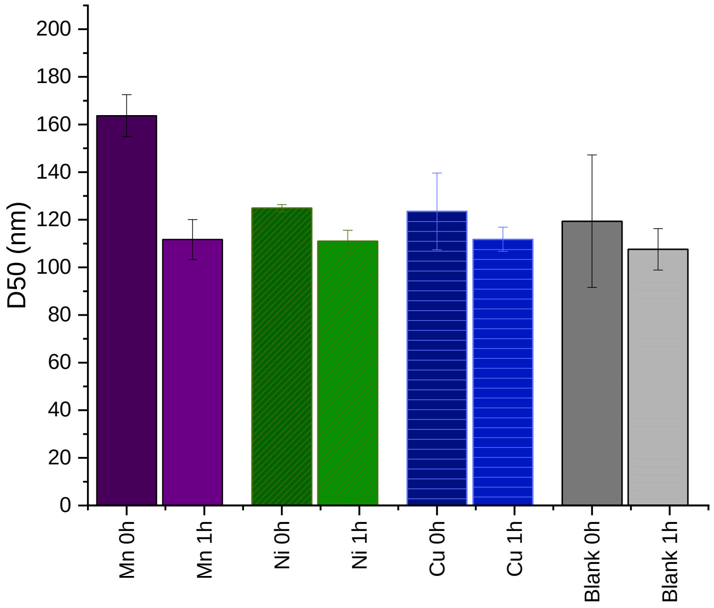

Figure S8. Particle size distributions determined by NTA for metal ions and PBS not significant particle size different with or without metal ions. HRP concentration was 8 u/mL. Metal ion concentrations added were the same as detected in metal release experiments with the same metal NPs.

References

1. Muilenberg, G.E. and C.D. Wagner, *Handbook of X-ray photoelectron spectroscopy : a reference book of standard data for use in X-ray photoelectron spectroscopy*. 1979, Eden Prairie, Minn.: Eden Prairie, Minn. : Perkin-Elmer.
2. Biesinger, M.C., et al., *Resolving surface chemical states in XPS analysis of first row transition metals, oxides and hydroxides: Cr, Mn, Fe, Co and Ni.* Applied Surface Science, 2011. **257**(7): p. 2717-2730.
3. Biesinger, M.C., *Advanced analysis of copper X-ray photoelectron spectra.* Surface and Interface Analysis, 2017. **49**(13): p. 1325-1334.
4. Rajamathi, M., P.V. Kamath, and R. Seshadri, *Polymorphism in nickel hydroxide: Role of interstratification.* Journal of Materials Chemistry, 2000. **10**(2): p. 503-506.
5. Krehula, S., et al., *Influence of Fe(III) doping on the crystal structure and properties of hydrothermally prepared β-Ni(OH)2 nanostructures.* Journal of Alloys and Compounds, 2018. **750**: p. 687-695.
6. Allahyar, S., et al., *Simple new synthesis of nickel oxide (NiO) in water using microwave irradiation.* Journal of Materials Science: Materials in Electronics, 2017. **28**(3): p. 2846-2851.

7. Aghazadeh, M., A.N. Golikand, and M. Ghaemi, *Synthesis, characterization, and electrochemical properties of ultrafine β-Ni(OH)2 nanoparticles.* International Journal of Hydrogen Energy, 2011. **36**(14): p. 8674-8679.
8. Ren, Y. and L. Gao, *From Three-Dimensional Flower-Like α-Ni(OH)2 Nanostructures to Hierarchical Porous NiO Nanoflowers: Microwave-Assisted Fabrication and Supercapacitor Properties.* Journal of the American Ceramic Society, 2010. **93**(11): p. 3560-3564.
9. Ashoka, S., G. Nagaraju, and G.T. Chandrappa, *Reduction of KMnO4 to Mn3O4 via hydrothermal process.* Materials Letters, 2010. **64**(22): p. 2538-2540.
10. Ishii, M., M. Nakahira, and T. Yamanaka, *Infrared absorption spectra and cation distributions in (Mn, Fe)3O4.* Solid State Communications, 1972. **11**(1): p. 209-212.
11. Li, Y., et al., *Synthesis of polyaniline nanotubes using Mn2O3 nanofibers as oxidant and their ammonia sensing properties.* Synthetic Metals, 2011. **161**(1): p. 56-61.
12. Ashoka, S., et al., *Synthesis and characterisation of microstructural α-Mn2O3 materials.* Journal of Experimental Nanoscience, 2010. **5**(4): p. 285-293.
13. Melendres, C.A., et al., *Synchrotron far infrared spectroscopy of surface films on a copper electrode in aqueous solutions.* Nuclear Inst. and Methods in Physics Research, B, 1997. **133**(1): p. 109-113.
